# Phenolic Compounds Profiling and Their Antioxidant Capacity in the Peel, Pulp, and Seed of Australian Grown Avocado

**DOI:** 10.3390/antiox12010185

**Published:** 2023-01-12

**Authors:** Xiaoyan Lyu, Osman Tuncay Agar, Colin J. Barrow, Frank R. Dunshea, Hafiz A. R. Suleria

**Affiliations:** 1 Faculty of Science, School of Agriculture and Food, The University of Melbourne, Parkville, VIC 3010, Australia; 2 Centre for Chemistry and Biotechnology, School of Life and Environmental Sciences, Deakin University, Waurn Ponds, VIC 3217, Australia; 3 Faculty of Biological Sciences, The University of Leeds, Leeds LS2 9JT, UK

**Keywords:** avocados, avocados peel, avocados pulp, polyphenols, phenolic compounds, phenolic acids, flavonoids, antioxidants, LC-MS, HPLC

## Abstract

Avocados (*Persea americana* M.) are highly valued fruits consumed worldwide, and there are numerous commercially available varieties on the market. However, the high demand for fruit also results in increased food waste. Thus, this study was conducted for comprehensive profiling of polyphenols of Hass, Reed, and Wurtz avocados obtained from the Australian local market. Ripe Hass peel recorded the highest TPC (77.85 mg GAE/g), TTC (148.98 mg CE/g), DPPH (71.03 mg AAE/g), FRAP (3.05 mg AAE/g), RPA (24.45 mg AAE/g), and ABTS (75.77 mg AAE/g) values; unripe Hass peel recorded the highest TFC (3.44 mg QE/g); and Wurtz peel recorded the highest TAC (35.02 mg AAE/g). Correlation analysis revealed that TPC and TTC were significantly correlated with the antioxidant capacity of the extracts. A total of 348 polyphenols were screened in the peel. A total of 134 compounds including 36 phenolic acids, 70 flavonoids, 11 lignans, 2 stilbenes, and another 15 polyphenols, were characterised through LC-ESI-QTOF-MS/MS, where the majority were from peels and seeds of samples extract. Overall, the hierarchical heat map revealed that there were a significant amount of polyphenols in peels and seeds. Epicatechin, kaempferol, and protocatechuic acid showed higher concentrations in Reed pulp. Wurtz peel contains a higher concentration of hydroxybenzoic acid. Our results showed that avocado wastes have a considerable amount of polyphenols, exhibiting antioxidant activities. Each sample has its unique value proposition based on its phenolic profile. This study may increase confidence in utilising by-products and encourage further investigation into avocado by-products as nutraceuticals.

## 1. Introduction

Avocado (*Persea americana* Mill.) is a member of the cinnamon family (Lauraceae) that originated in the tropical areas of America and planted and cultivated in the neotropics since 10,000 BC [1]. It was introduced to Australia in the late 19th century [2], and it has since grown in popularity, becoming an essential fruit in Australia. Hass, Hazzard, Pinkerton, Gwen, Fuerte, Wurtz, Reed, and Shepard are widely grown varieties in Australia [3]. Avocados possess high nutritional value and contain bioactive compounds, including fibre, phenolic compounds, vitamins B and E, and carotenoids, which positively impact human health [4]. A considerable number of avocados are processed into avocado products, such as guacamole sauce, avocado pulp powder for pasta, and more, in addition to being eaten as fresh fruit. Processing, retail, and distribution are projected to generate 20% of total food waste [5], and avocado industrial processing produces a significant amount of peels and seed waste. Avocado waste produced in industrial processing could be a potential source of antioxidants and other biologically active substances. Previously, Wang, et al. [6] stated that avocado waste from processing could be used in the nutraceutical industry, increasing the potential value of avocado residues and leading to innovative functional food development.

Phenolic compounds are secondary plant metabolites that generally exist in the tissues of plants, in which their types and contents are significantly different with plant varieties, maturity, seasons, and regions [7]. Each variety of fruit has its own complexity and characteristics in terms of the composition and content of phenolic compounds [8]. Phenolic compounds possess excellent antioxidant potential due to their high redox reactivity to reduce free radicals and prevent destructive cascade reactions. It can strengthen blood vessel walls, promote digestion, reduce blood lipid, enhance human immunity, prevent arteriosclerosis and thrombosis, reduce diuresis, lower blood pressure, and prevent the proliferation of bacteria and cancer cells. This signifies the potential value of exploiting polyphenol-rich food such as avocados for other industries.

Phenolic estimation of avocado in pulp, peel, and seed can be achieved by total phenol content (TPC), total flavonoid content (TFC), and total tannins content (TTC). Furthermore, the identification characterisation of phenolic compounds in avocados can be achieved by liquid chromatography electron spray quadrupole tandem mass spectrometry (LC-ESI-QTOF-MS/MS) and high-performance liquid chromatography coupled with photodiode array detector (HPLC-PDA) techniques. Previously, some phenolic compounds such as chlorogenic acids, epicatechins, and catechins were characterised in avocado peels using liquid chromatography-mass spectrometry analysis in past studies [9] but most studies focus on measurement of phenolic compounds in pulp; therefore, comprehensive profiling of phenolic compounds in Australian grown avocados remains in shadow. Thus, this study aims to identify, characterise, and quantify the phenolic compounds of avocado in the pulp, seed, and peel. The results of this study may positively influence the avocado and food processing industries, encouraging the exploration of novel applications and adding value to avocado products.

## 2. Materials and Methods

### 2.1. Chemicals and Reagents

Most of the reagents, chemicals, and standards used for extraction and characterisation were analytical grades. Ethanol, methanol, and gradient grade acetonitrile were purchased from Merck KGaA (Darmstadt, Germany). Folin–Ciocalteu’s phenol reagent, gallic acid, sodium carbonate, aluminium chloride, quercetin, vanillin, catechin, 2,2-diphenyl-1-picrylhydrazyl (DPPH), ascorbic acid, 2,4,6-tris(2-pyridyl)-s-triazine (TPTZ), iron [III] chloride, L-ascorbic acid, acetic acid, and potassium persulfate were purchased from Sigma-Aldrich (Castle Hill, NSW, Australia). Sodium acetate was purchased from Thermo Fisher Scientific (Sunnyvale, CA, USA). Sulphuric acid was purchased from RCI Labscan Limited (Bangkok, Thailand). 2,2′-azino-bis (3-ethylbenzothiazoline-6-sulfonic acid) (ABTS) was acquired from Roche Diagnostics GmbH (Nunawading, VIC, Australia). HPLC grade standards including catechin, quercetin 3-*O*-galactoside, quercetin 3-*O*-glucuronide, kaempferol 3-*O*-glucoside, quercetin, kaempferol, protocatechuic acid, p-hydroxybenzoic acid, chlorogenic acid, caffeic acid, syringic acid, coumaric acid, and ferulic acid were purchased from Sigma-Aldrich (St. Louis, MO, USA). Water was deionised to reach a resistivity of 18.2 MΩ/cm using a Millipore Milli-Q Gradient Water Purification System (Darmstadt, Germany) and was filtered through a 0.45 µm type Millipak^®^ Express 20 Filter (Milli-Q, Darmstadt, Germany).

### 2.2. Collection of Sample

Three different varieties of avocado fruit, Hass, Reed and Wurtz, were purchased from the local market (Victoria Market). These avocados were randomly collected and selected for their firmness, absence of mechanical damage, and lack of visible decay. First, all samples were manually cleaned, then pulp, peels, and seeds were separated manually, cut into small pieces, and blended into slurries (Russell Hobbs Classic, model DZ-1613, Braeside, VIC, Australia). After that, samples were stored in a −20 °C freezer.

### 2.3. Extraction of Polyphenols

We extracted 5 g of pulp, peel, and the seed of the three avocado varieties with 20 mL of 80% (*v*/*v*) ethanol and homogenised for 30 s with the Ultra-Turrax T25 Homogenizer (Jane & Kunkle IKA-Labortechnik, USA). Then, all samples were incubated at 120 rpm at 4 °C in a shaking incubator (ZWYR-240, Labwit, Ashwood, VIC, Australia) for 12 h. Then, samples were centrifuged at 5000 rpm at 4 °C for 15 min in a benchtop centrifuge (Zentrifugen Rotina 380R, Hettich, Germany). Then, the supernatant was collected and filtrated through 0.45 μm syringe filter (Thermo Fisher Scientific Inc., Waltham, MA, USA) for further analysis.

### 2.4. Estimation of Phenolic Compounds and Antioxidant Assays

The Phenolic estimation (TPC, TFC, and TTC) and antioxidant assay (DPPH, FRAP, ABTS, RPA, ·OH-RSA, FICA and TAC) were carried out according to the method of Tang, et al. [10]. Each sample was analysed in triplicate, and absorption data were measured by the Multiskan^®^ Go microplate photometer (Thermo Fisher Scientific, Waltham, MA, USA). The standard curves were plotted with R2 > 0.995.

#### 2.4.1. Total Phenolic Content (TPC)

The total phenolic content was measured according to Severo, et al. [11] with minor modifications. We added 25 µL of extract, 25 µL of Folin–Ciocalteu reagent solution (1:3 diluted in water), and 200 µL of water into the 96-well plate. Then, the reaction mixture was incubated for 5 min in the dark at 25 °C. Then, 25 µL of 10% (*w*/*w*) sodium carbonate was added to the reaction mixture and incubated for 60 min at 25 °C. The absorbance was measured at 764 nm. Gallic acid standard with concentrations from 0 to 200 µg/mL was constructed to prepare the standard curve. Results were expressed as mg of gallic acid equivalents (GAE) per gram of a sample.

#### 2.4.2. Total Flavonoid Concentration (TFC)

The TFC was quantified by using the aluminium chloride method of Danying, et al. [12] with minor modification. We added 80 µL of extract, 80 µL of 2% aluminium chloride, and 120 µL of sodium acetate solution into the 96-well plate. The mixture was incubated for 2.5 h in the dark at 25 °C. The absorbance was measured at 440 nm, and a quercetin calibration curve with 0–50 µg/mL was constructed to estimate TFC. Results were expressed as mg of quercetin equivalents (QE) per gram of a sample.

#### 2.4.3. Determination of Total Tannins Concentration (TTC)

The TTC was performed based on the method of Zou, et al. [13] with modifications. We added 20 µL of extract, 150 µL of 4% vanillin solution, and 25 µL of 32% (*v*/*v*) sulphuric acid into the 96-well plate, and it was incubated for 15 min in the dark at 25 °C; then, absorbance was measured at 500 nm. Catechin standard curve ranging from 0 to 1000 µg/mL was constructed to estimate TTC. The results were expressed as mg of catechin equivalent (CE)/g of weight from samples.

#### 2.4.4. 2,2′-Diphenyl-1-picrylhydrazyl (DPPH) Assay

The DPPH assay was performed in reference to Hasan, et al. [14] with some modifications. We added 40 µL of the extract to 260 µL of 0.1 mM DPPH solution in a 96-well plate, and it was incubated for 40 min in the dark at 25 °C. Absorbance was measured at 517 nm. The ascorbic acid calibration curve with concentrations ranging from 0 to 50 µg/mL was constructed to determine the DPPH value and expressed in mg of ascorbic acid equivalent per gram (mg AAE/g) of a sample.

#### 2.4.5. Ferric Reducing Antioxidant Power (FRAP) Assay

Based on Benzie and Strain [15] method with minor modification, FRAP reagent was prepared daily, in the volume ratio 10:1:1, 300 mM acetate buffer (pH 3.6) with 10 mM TPTZ and 20 mM FeCl_3_ was mixed to prepare FRAP dye solution. We added 20 μL of the extract and 280 μL of FRAP solution to a 96-well plate, and it was incubated for 5 min at 37 °C, and absorbances were measured at 593 nm. The ascorbic acid calibration curve with concentrations ranging from 0 to 150 µg/mL was used to determine the FRAP value and expressed in mg of ascorbic acid equivalent per gram (mg AAE/g) a sample.

#### 2.4.6. 2,2′-Azino-bis-3-Ethylbenzothiazoline-6-Sulfonic Acid (ABTS) Assay

The ABTS free radical scavenging activity of samples was estimated using the method of Tang, et al. [10] with some modification. The ABTS stock solution was prepared by mixing of 5 mL of 7 mM ABTS solution and 88 μL of 140 mM potassium persulfate, incubated at room temperature for 16 h. Then, the stock solution was diluted with ethanol, 10 μL of extract and 290 μL of the ABTS solution were mixed, and then it was incubated at 25 °C for 6 min in the dark. Absorbance was measured at 734 nm. The ascorbic acid calibration curve with concentrations ranging from 0 to 150 µg/mL was used to determine the ABTS value and expressed in mg of ascorbic acid equivalent per gram (mg AAE/g) a sample.

#### 2.4.7. Reducing Power Assay (RPA)

The reducing power activity was determined by the method of Ferreira [16], with modifications. We added 10 μL of sample extract, 25 μL of 0.2 M sodium phosphate buffer (pH 6.6), and 25 μL of K_3_[Fe(CN)_6_], then, the mixture was incubated for 20 min at 25 °C. We then added 25 μL of TCA solution (10%) to stop further reaction, followed by the addition of 85 μL of water and 8.5 μL of FeCl_3_, and it was incubated further for 15 min at 25 °C. Absorbance readings were measured at 750 nm, and a standard curve from ascorbic acid (0 to 500 µg/mL) was prepared. Results were expressed as mg AAE/g.

#### 2.4.8. Hydroxyl Radical Scavenging Activity (·OH-RSA) Assay

The hydroxyl radical scavenging activity of samples was estimated by using the Smirnoff [17] method with modifications. We added 50 μL of sample extract to the combination mixture of 50 μL of 6 mM FeSO_4_·7H_2_O and 50 μL of 6 mM H_2_O_2_ (30%), which was then incubated for 10 min at 25 °C. Subsequently, 50 μL of 6 mM 3-hydroxybenzoic acid was added. Absorbance readings were measured at 510 nm, with a standard curve prepared from ascorbic acid (0–300 µg/mL). Results were expressed as mg AAE/g.

#### 2.4.9. Ferrous Ion Chelating Activity (FICA) Assay

A modified method on Dinis [18] was used to determine chelating activity of ferrous ions. The solution mixture was made up of 15 μL of sample extract, 85 μL of water, 50 μL of 2 mM ferrous chloride (1:15 water dilution), and 50 μL of 5 mM ferrozine (1:6 water dilution), which was incubated for 10 min at 25 °C. Absorbance was measured at 562 nm, and a standard curve was generated from Ethylenediaminetetraacetic acid (EDTA), ranging from 0 to 30 μg/mL. Results were expressed as mg EDTA/g.

#### 2.4.10. Total Antioxidant Capacity (TAC)

Referring to Jan, et al. [19], the total antioxidant capacity of samples was conducted using the phosphomolybdate method. The sulphuric acid (0.6 M), 28 mM sodium phosphate, and 4 mM ammonium molybdate were mixed to form a TAC dye solution. We added 40 μL of extract and 260 μL of dye solution to the 96-well plate, and it was incubated in a water bath at 95 °C for 90 min. After the samples were cooled, the absorbance of the mixture was measured at 765 nm. The ascorbic acid calibration curve with concentrations ranging from 0 to 200 µg/mL was used to determine the TAC value and expressed in mg of ascorbic acid equivalent per gram (mg AAE/g), of a sample.

### 2.5. Identification and Characterization of Phenolic Compound by LC-ESI-QTOF-MS/MS

The LC-ESI-QTOF-MS/MS analysis was performed based on the study by Suleria, et al. [20]. Agilent 1200 series HPLC (Agilent Technologies, Santa Clara, CA, USA) equipped with an Agilent 6520 Accurate-Mass QTOF LC/MS (Agilent Technologies, Santa Clara, CA, USA) was used for the identification and characterization of polyphenols form avocado. The separation was carried out using a Synergi Hydro-RP 80A, LC column 250 × 4.6 mm (Phenomenex, Torrance, CA, USA). Mobile phase A was prepared in the ratio of water/acetic acid (99.5:0.5 *v*/*v*), and mobile phase B consisted of acetonitrile/water/acetic acid (50:49.5:0.5, *v*/*v*/*v*). Both mobile phases A and B were degassed at 21 °C for 15 min. The extract was filtered using Syringe Filters (Kinesis Australia, Redland, QLD, Australia), then transferred into vials. The flow rate was set at 0.8 mL/min, and the injection volume was 5 μL. ESI was used to allow operation in both negative and positive modes. Mass spectra in the *m*/*z* ranged from 50 to 1300. The mass spectrometry conditions were set as follows: nitrogen gas temperature at 300 °C with a flow rate of 5 L/min, sheath gas temperature of 250 °C with a flow rate of 11 L/min, and nebuliser gas pressureof 45 psi. The capillary and nozzle voltage were set at 3.5 kV and 500 V, respectively. Data acquisition and analysis were performed using Agilent Mass Hunter Data Acquisition Software Version B.03.01.

### 2.6. Quantification of Phenolic Compounds by HPLC—PDA

The quantification of targeted phenolic compounds present in avocado was carried out by Agilent 1200 series HPLC (Agilent Technologies, Santa Clara, CA, USA) equipped with a PDA following the Zhong, et al. [21] method. The same column and conditions were maintained as described in LC-ESI-QTOF-MS/MS protocol except for the sample injection of 20 μL. Detection was examined at three different wavelengths (280 nm, 320 nm, and 370 nm) for various phenolic compounds. Data acquisition and analysis were performed using Agilent Mass Hunter Data Acquisition Software Version B.03.01.

### 2.7. Statistical Analysis

Phenolic estimation and antioxidant capacity of phenolic compounds of avocado were analysed by one-way analysis of variance (ANOVA) through Minitab Version 19.0 (Minitab, LLC, State College, PA, USA) using the setting Fisher’s least significant difference (LSD) test at *p* < 0.05. The data were presented as the mean ± standard deviation.

## 3. Results and Discussion

### 3.1. Estimation of Phenolic Compounds (TPC, TFC and TTC)

According to the results of the TPC, TFC and TTC were performed to determine the phenolic content. It was observed that all avocados studied were quite rich in polyphenols (Table 1). In our study, the highest concentration of TPC was present in avocado peel of Hass (ripe) with 77.85 mg GAE/g. TPC of seed samples ranged from 26.93 to 44.91 mg GAE/g, and the ranges for peel and pulp were 29.22–77.85 mg GAE/g and 0.20–0.28 mg GAE/g, respectively. Wang, et al. [6] determined the TPC of avocado seeds, peels, and pulps in several varieties and presented that the TPC of Hass avocado’s seed, peel, and pulp were 51.6, 12.6, and 4.9 mg GAE/g, respectively. Rodríguez-Carpena, et al. [22] reported TPC of fully ripened Hass peel and seed extracts as 89.97 GAE mg/g and 60.82 mg GAE/g obtained by an acetone/water blend. While Calderón-Oliver, et al. [23] reported 5.7 mg and 19.7 mg GAE/g in Hass seed and peel extracts, which is much lower than the results from this study. In another study, avocado dried peel showed lower TPC with 1252.31 ± 165.62 mg GAE 100 g^−1^ compared to our study [24]. Wang, et al. [6] showed that the TPC value of Hass seed was around three times more than that of Hass peel, which is different from this study. Also, the TPC of pulps from previous studies yielded higher results than this study. As can be seen, in spite of the fact that different studies have been carried out on the determination of phenolic content in avocado species before, some differences can be observed due to factors such as geographical growth location, ripeness, climate, storage conditions, and the extraction solvents used. As a result, it is critical for public health, as was the case in the current study, to investigate the content of phenolic compounds in nutrients found in local markets, which are widely used by the general public for many different purposes. In addition, although the Folin–Ciocalteu reagent has been successfully applied for the determination of total phenolic compounds for many years, it can also give positive results with many nonphenolic compounds such as some vitamins and elements [25]. For this reason, it should not be forgotten that, in addition to the total phenol content, it is important to determine the individual phenolic compounds as in this study.

Flavonoids are a large class of natural products widely found in the plant kingdom. Most flavonoids exist as glycosylated derivatives in the plant (for example, combination with glucose or rhamnose), and some of them are in the free state or exist in combination with tannins [26]. The aluminium chloride (AlCl_3_) colorimetric method commonly measures TFC. In this study, a higher concentration of TFC was observed in Hass (unripe) avocado peel with 3.44 mg QE/g. The ranges of TFC of seed, peel, and pulp were 0.06–2.75 mg, 0.38–3.44 mg, and 0.01–0.09 mg QE/g, respectively. Morais, et al. [24] reported that TFC value for avocado seed and peel were 0.3 and 1.56 mg quercetin equivalent (QE)/g, respectively. Similarly, Shehata and Soltan [27] reported that TFC value of avocado seed and pulp were 3.21 and 2.96 mg QE/100 g, respectively. TFC value of seed and peel in Hass and Wurtz were closer to the study of Morais, et al. [24]. Amado, et al. [28] measured 0.51 mg QE/g TFC of Wurtz seed from Riyadh, which is higher than shown in this study. The extraction time and temperature might be the factors that caused higher results. In previous studies, the concentration of flavonoids tended to decrease as the fruit progressively ripened [29].

The consumption of condensed tannins-rich foods can decrease cancer incidence because of the antioxidative property [30]. Ripe Hass peel contained the highest tannins content (148.98 mg CE/g) among the samples, and both seeds and peels are a favourable source of tannins based on our results. Ge, et al. [31] reported that the two varieties of Chinese avocado pulp has almost no tannins, which is consistent with our results. Moreover, the stage of maturity of the avocado fruit can influence the TPC, TFC, and TTC value. The polyphenol in Reed is abundant, but the study and literature about Reed avocado have been extremely limited, so this work contributed by increasing knowledge of it for further research.

### 3.2. Antioxidant Activity

It is not enough to use just one method to determine the antioxidant activity of natural compounds, since antioxidant activity affects many mechanisms, such as repairing biological damage, sequestering transition metal ions, and scavenging free radicals. It is essential to apply methods that work with different mechanisms simultaneously in order to understand the full picture [32]. Factors including solvent, temperature, the chemical structure of phenolic compounds, and pH can influence the antioxidant mechanism and affect the accuracy of estimating the antioxidant activity. Thus, more than one method was deployed to evaluate the antioxidant activity of samples. ABTS, DPPH, FRAP, RPA, FICA, ·OH-RSA, and TAC assays are widely used colorimetric methods for the determination of antioxidant capacity, and they do not require complicated testing equipment to operate [25].

DPPH is a stable free radical that can be used to test the ability of the sample’s polyphenols to scavenge DPPH free radicals. In our study, Wurtz seed observed significantly stronger DPPH scavenging ability (56 mg AAE/g) than other seeds. DPPH values of peels from Hass (ripe) and Wurtz were 71.03 and 66.13 mg AAE/g, respectively, which are significantly higher than that of Reed and unripe Hass. This result reflects the Wang et al. [6] statement of that Hass peel possessed higher DPPH value than that of Hass seed, and the value of the pulp was approximately 150 times less than that of the seed. In addition, ripe Hass seeds exhibited higher scavenging activity than the unripe Hass, which is consistent with a previous study [33].

The principle of FRAP assay was to reduce the colorless Fe^3+^–TPTZ complex to produce blue-colored Fe^2+^–TPTZ complex under low pH condition by antioxidants present in the sample extract. In our study, the FRAP values of seeds are higher than that of other parts, and Wurtz seed yielded the highest value (3.69 mg AAE/g). FRAP antioxidant capacity of ripe Hass peel (3.05 mg AAE/g) was higher than that of ripe Hass seed (0.98 mg AAE/g). In Wurtz and Reed avocado, Morais, et al. [24] reported that freeze-dried seed presented the highest antioxidant capacity compared with raw pulp and freeze-dried peel.

ABTS is used as a chromogenic agent, which is oxidised by active oxygen to form a stable blue-green cation free ABTS^+^. Avocado peel showed higher ABTS antioxidant activity than seed and pulp, and the value of ripe Hass peel was 75.77 mg AAE/g, which was the highest among the extract samples. There was no significant difference in the antioxidant activity among pulp sample extract. The level of antioxidant activity observed in seed and peel of Reed and unripe Hass were also not significantly different (*p* < 0.05). Ripe Hass possessed the greatest ABTS radical scavenging capacity (74.14 mg AAE/g), significantly higher than seed of Wurtz, Reed, and unripe Hass. Ortega-Arellano, et al. [34] reported that antioxidant activity for Hass peel was greater than that of Reed peel, which is consistent with our result. Also, no significant difference in the ABTS antioxidant activity among the pulps of different avocado varieties was found.

In our study, Hass (ripe) avocado peel exhibited stronger reducing power (24.45 mg AAE/g) than the seed and pulp. Reed pulp displayed the lowest reducing power (0.17 mg AAE/g). Findings from previous studies corroborate with our study whereby the trend follows: peel > seeds > pulp [35,36]. Although, Wurtz seed (14.28 mg AAE/g) was comparable to the reducing power of the different peel varieties. Avocado pulp is known to exhibit relatively lower reducing power [37].

Avocado seed had higher values for hydroxyl radical scavenging activity, especially in the Reed variety (13.25 mg AAE/g). However, pulp showed less scavenging ability with values ranging from 0.14–1.14 mg AAE/g. Oboh, et al. [38] revealed that the avocado seed effectively scavenging for hydroxyl radicals. Interestingly, they found pulp to be more effective than peel.

The ability to chelate transition metal ions is used as an antioxidant determinant, as these transition metals promote and propagate radical generation. In our study, avocado seed, particularly from the Reed variety (9.68 mg EDTA/g), produced the most chelating activity, followed by peel, and ultimately the lowest produced by ripe Hass pulp (0.17 mg EDTA/g). Oboh, Adelusi and Akinyemi [38] also found similar results to our study. The presence of the following functional groups: –S–, –O–, –OH, –SH, –COOH, PO_3_H_2_, C=O, –NR_2_, have previously been reported to contribute to metal chelating activity [39].

The principle of measuring total antioxidant capacity is that molybdenum (VI) is reduced to molybdenum (V) complex by antioxidants, which turns the solution green. In this study, the highest TAC value was observed in Wurtz peels with 35.02 mg AAE/g, and the lowest was observed in Hass unripe pulp with 0.25 mg AAE/g. The order of TAC for seeds was ripe Hass (27.49 mg AAE/g) > Wurtz (19.48 mg AAE/g) > unripe Hass (13.26 mg AAE/g) > Reed (6.58 mg AAE/g). Alkhalaf, et al. [40] showed that the total antioxidant capacity of the avocado seed was much greater than that of pulp, which is consistent with our results. Folasade, et al. [41] indicated that the TAC for avocado seed ranged from about 1.7 to 2.6 mg AAE/g based on different extraction solvents. Furthermore, Duresa [42] measured the TAC for edible portions of avocado from three different districts and produced results of 0.292, 0.274, and 0.265 mg AAE/25 g, which are lower than our results. The assays’ results showed that seed and peel’s antioxidant activities were much higher than that of pulp. Moreover, Alagbaoso, Tokunbo and Osakwe [33] reported that ripe avocado seed possessed a stronger antioxidant capacity than unripe avocado, which supports our results.

### 3.3. Correlation of Polyphenols and Antioxidant Activities

In the correlation analysis (Table 2), a high positive strong correlation (0.70 < r < 0.90, *p* < 0.01) was observed between total phenolic content and antioxidant activity (TTC, DPPH, FRAP, ABTS, RPA, and TAC) and may be attributed to the rich variety of phenolic compounds in avocado that act as hydrophilic antioxidants [43,44]. It is highlighted that the correlation between DPPH radical-scavenging activity and TPC had significant correlation (r = 0.964, *p* < 0.05). This high correlation suggests that phenolic compounds were the main contributors to the antioxidant activity measured in avocados. The relationship between phenolic compounds content and the radical scavenging capacities in avocados was consistent with Dudonné, et al. [45].

In this study, TFC value does not show a correlation with other assays. On other hand, TTC showed the same trend with TPC, exhibiting high correlation with antioxidant activity, especially with DPPH (r = 0.851, *p* < 0.01), ABTS (r = 0.814, *p* < 0.01), and RPA (r = 0.891, *p* < 0.01). The significant correlations (*p* < 0.05) between DPPH, FRAP, ABTS, and RPA were found, especially between DPPH and ABTS (r = 0.910, *p* < 0.01), and RPA. The proposed reason for this observation is that the redox reactions of these assays are similar [25]. This suggests that phenolic compounds in avocado can effectively scavenge radicals and chelate transition metals. In addition, the fact that phenolic compounds show activities such as hypolipidemic, hypercholerostemic, anti-obesity, acetylcholine esterase inhibitor in many in vivo studies [46] supports the strong radical scavenging effect in our in vitro findings.

### 3.4. Distribution of Polyphenols—Venn Diagram

**Figure 1:**
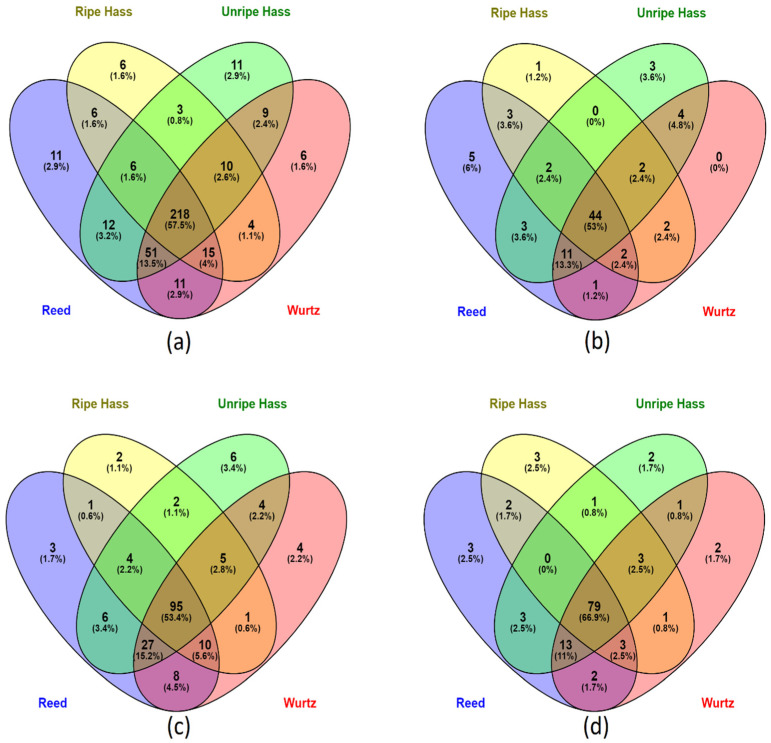
Venn diagram of screened phenolic compounds species present in various avocado varieties. (**a**) distribution of all the screened phenolic compounds in all avocado parts (peel, pulp and seed) from the four varieties. (**b**) distribution of phenolic acids in all parts of the four avocado varieties. (**c**) distribution of flavonoids in all parts of the four avocado varieties. (**d**) distribution of other polyphenols (including lignans and stilbenes) in all parts of the four avocado varieties.

A total of 379 compounds were screened from the avocado samples (Figure 1a); most of the polyphenols (57.5%) were common throughout all the varieties. The Reed and Wurtz samples had a higher diversity of phenolic compounds, with 87% and 85 %, respectively. Ripe Hass represented the least diversity of phenolic compounds in its parts, with approximately 70% of the phenolic compounds.

**Figure 2:**
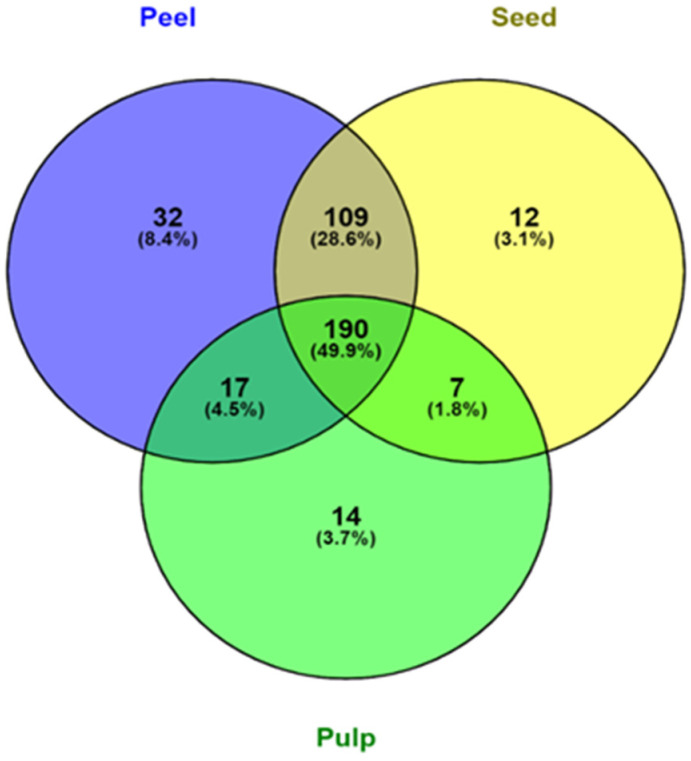
Venn diagram representation of the distribution of phenolic compounds in peel, pulp, and seed samples of the four varieties of avocados.

A total of 83 phenolic compounds were identified and screened, where 53% of phenolic acids were common across all sample extracts (Figure 2a). In comparison, Reed had the highest diversity of phenolic acids, containing about 85%, and 6% of phenolic acids were unique to Reed’s profile. Ripe Hass consist of the least diversity of phenolic acids, containing only 67% of all the screened phenolic acids. Interestingly, Wurtz did not contain any unique phenolic acids. In flavonoids, unripe Hass represented 3.4% of flavonoids that were unique to its profile, highest among all varieties, followed by Wurtz, which observed 2.2% of flavonoids unique to its profile as shown in Figure 1c. Reed and Wurtz both contain 86% of the screened 178 flavonoid compounds and share 90% of their flavonoid profile, whereas ripe Hass only contained roughly 67% of the screened flavonoids, the lowest among all varieties. All varieties share 66.9% of their other polyphenol profile with each other as displayed in Figure 1d. Again, Reed contained the highest diversity of other polyphenols (about 89%), followed closely by Wurtz (88%) and unripe Hass (86%), with ripe Hass containing the lowest diversity (77%). Overall, the collective Reed samples screened out more polyphenols than other varieties. Additionally, there were more polyphenol varieties in unripe Hass than ripe Hass samples.

Out of all the screened polyphenols, 348 were found in avocado peels (Figure 2). Avocado peels contain the most phenolic compounds, followed by seed and pulp. 78.5% of all screened phenolic compounds were found in both peel and seed, accounting for a substantial portion of their phenolic profile. The avocado peel also contained a significant portion of unique phenolic compounds with 8.4%. This finding is consistent with the result from Table 1 and the findings from Rodríguez-Carpena, et al. [47], where avocado peel and seed contained more varieties of polyphenols than the pulp, which consequently led to the higher antioxidant activity in peel and seed samples. Leaf, peel, and seed of avocados have been found to contain most of the polyphenols, whereas carotenoids, sterols, and tocopherols were found exclusively in the pulp [48]. Therefore, there may be value in rescuing and utilising the peel and seed of avocado instead of throwing them away as waste.

### 3.5. Characterization of Polyphenols

A total of 134 phenolic compounds were tentatively characterised from the sample extract through LC-ESI-QTOF-MS/MS technique. The data were summarized in Table 3, with all compounds selected that were less than 5 ppm. Compounds were classified as phenolic acids, flavonoids, lignans, stilbenes, and other polyphenols. In addition, LC-ESI-QTOF-MS/MS basic peak chromatograph (BPC) for characterization of phenolic compounds of avocados can be found in Figure S1 in Supplementary Materials.

#### 3.5.1. Phenolic acids

A total of 36 phenolic acids were identified including (10) hydroxybenzoic acids, (21) hydroxycinnamic acids, (2) hydroxyphenyl acetic acids, and (3) hydroxyphenyl propanoic acids. Compound 2 was found in both modes of ionisation and tentatively identified as 2,3-dihydroxybenzoic acid based on its *m*/*z* at 153.0195 with product ion at *m*/*z* 109. It was present in the seed of the ripe Hass, unripe Hass, and Reed sample. Compound 6 was tentatively identified as galloyl glucose, which exhibited a peak at [M-H]^−^ *m*/*z* 331.0682 and produced fragments at *m*/*z* 169 and *m*/*z* 125. Galloyl glucose was found in all avocado varieties except for unripe Hass. A study on Albizia anthelminthica leaf extract also observed similar fragmentation pattern, with the product ions at *m*/*z* 169 and *m*/*z* 125 corresponding to the sequential loss of glucose moiety and carboxyl group, respectively [49]. A galloyl glucose was also found in the Rhus typhina stem extract [50].

Compound 17 was found in a peak at [M-H]^−^ *m*/*z* 353.0875 and fragmented at *m*/*z* 253, *m*/*z* 190, and *m*/*z* 144 was tentatively identified as 3-caffeoylquinic acid and was observed in all samples except ripe Hass pulp and Wurtz pulp. In addition, compound no. 31, 1,5-dicaffeoylquinic acid at [M-H]^−^ at *m*/*z* 515.1212 was also observed in Reed seed, Reed peel, ripe Hass peel, and Wurtz pulp. In the study conducted by Kosinśka et al. on Hass peels and seeds, 5-*O*-caffeoylquinic acid and 3-*O*-caffeoylquinic acid were observed, respectively, whereas 1,5-dicaffeoylquinic acid was not observed [35]. Our study showed that the Hass variety had a richer profile of caffeoylquinic acid, which has anti-inflammatory and antioxidant properties [51].

Compound 28 with a precursor ion at [M-H]^−^ *m*/*z* 223.0613 produced product ions at *m*/*z* 205 and *m*/*z* 163, which was tentatively identified as sinapic acid. It was present in both ionisation modes and all varieties but not in the unripe Hass pulp. Previously, Rosero, et al. [52] also identified sinapic acid in the peel and seed of the Nariño avocado cultivar. The sinapic acid in our study was observed as an aglycone, whereas Lopez-Cobo et al. observed a glycoside derivative, sinapic acid-C-hexoside, in their study [53]. Sinapic acid is found in many different fruits, vegetables, herbs, and cereals, and it possesses DPPH and superoxide antioxidant activity.

#### 3.5.2. Flavonoids

Seventy flavonoids were identified from the samples, including anthocyanins (9), dihydrochalcones (2), dihydroflavonols (3), flavanols (10), flavanones (6), flavones (9), flavonols (17), and isoflavonoids (14). Among the anthocyanins, all the identified compounds were glycosylated. Based on the MS spectrum, Compound 38 was found in positive ionisation mode at *m*/*z* of 628.1630, and Compound 42 was found in positive ionisation mode at *m*/*z* 450.1143. They were tentatively identified as delphinidin 3-*O*-glucosyl-glucoside and cyanidin 3-*O*-galactoside, respectively. Both compounds were only found in avocado peels and seeds from all four varieties. According to Prabha, et al. [54], cyanidin 3-*O*-galactoside, also known as ideas, was the major anthocyanin in the avocado peel to develop the colour, especially for the ripened avocado peel. Compound 41 produced peak at [M+H]^+^ *m*/*z* 466.1098 and fragment ion at *m*/*z* 303, which led to the tentative identification of the compound as delphinidin 3-*O*-glucoside. A previous study on anthocyanin content of strawberry fruit in different extraction conditions also reported the same fragment ion for delphinidin 3-*O*-glucoside, in which the fragment was a result of the loss of glucose moiety [55].

Compound 69 was found in Reed and ripe Hass peel and Wurtz pulp and was tentatively identified as gardenin B, with product ions produced at *m*/*z* 344, *m*/*z* 329, and *m*/*z* 311. This is the first time that gardenin B was characterised in avocado to the best of our knowledge. A previous study on Ocimum leaf extract also obtained the same fragmentation pattern [56]. Furthermore, a study has shown that gardenin B induces apoptosis in human leukaemia cells [57]. The presence of such a beneficial compound to human health in avocado peels increases the avocado by-product’s value for further exploitation rather than for disposal.

#### 3.5.3. Lignans

In this study, 11 lignans were detected in avocado samples. Compound 113 found in both ionisation modes produced a peak at [M-H]^−^ *m*/*z* 357.1348 and fragmented at *m*/*z* 342, *m*/*z* 327, *m*/*z* 313, and *m*/*z* 221. This compound was tentatively assigned as matairesinol and was only found in the peels and seeds of Reed, unripe Hass, and Wurtz samples. A similar fragment pattern was also observed by Eklund, et al. [58], with product ion peaks at *m*/*z* 342, *m*/*z* 313, *m*/*z* 298, and *m*/*z* 209 for matairseinol via MS/MS tandem mass spectrometry. Matairesinol has previously been identified in avocado as well as other fruits, vegetables, and herbs [59]. It has been reported that this compound is readily converted by intestinal microbes into enterolactone, a mammalian lignan with estrogenic activity [60].

#### 3.5.4. Stilbenes

Based on the MS spectra, two stilbenes were characterised in a sample. Compound 118, identified as resveratrol, was found only in the peels and seeds of the avocados. The fragmentation produced peaks at *m*/*z* 212, *m*/*z* 185, *m*/*z* 157, and *m*/*z* 143, and this pattern has also been observed in a past tandem mass spectrometric analysis of resveratrol [61]. Resveratrol has been extensively studied for its reported health benefits and effective antioxidant capacities, anti-inflammatory properties, and potentially anti-cancer effects, which has led to the successful commercialisation of resveratrol as nutraceutical products [62].

#### 3.5.5. Other Polyphenols

Apart from the above phenolic compounds, 15 other polyphenols were identified in these samples, including alkylmethoxyphenols (1), furanocoumarins (1), hydroxybenzaldehydes (1), hydroxybenzoketones (1), hydroxycoumarins (4), hydroxyphenylpropenes (1), other polyphenols (1), phenolic terpenes (1), and tyrosols (4). Compound 127 was primarily found in the peel and seed samples. The compound was present in [M-H]^−^ at *m*/*z* 191.0358 and produced a fragment at *m*/*z* 176, leading to the tentative identification of this compound as scopoletin. Scopoletin is a hydroxycoumarin, which has been previously characterised in avocados and has been studied for its potential health benefits as it modulates several cell signalling pathways [63].

### 3.6. Heatmap and Hierarchical Cluster Analysis Phenolic Compounds

**Figure 3:**
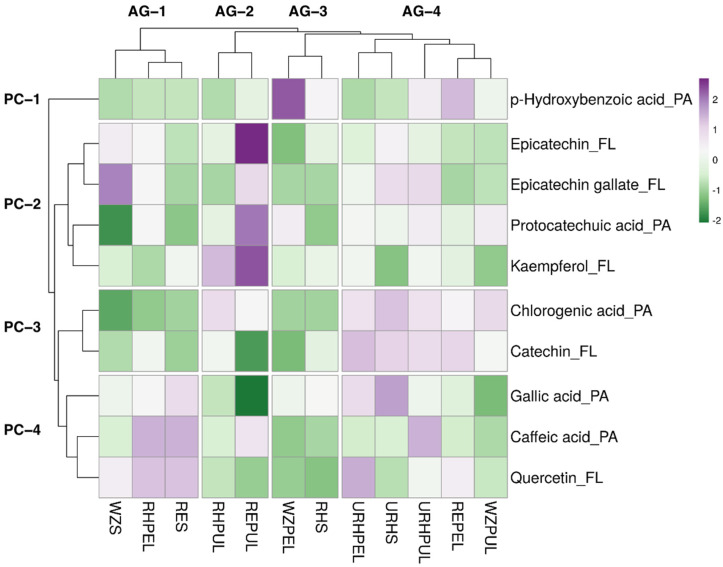
Heat map of the distribution of 10 selected phenolic compound in the avocado samples. Increase in purple coloration indicates higher average concentration of the corresponding phenolic compound in the corresponding sample, whereas increase in green coloration indicates lower average concentration. AG: avocado sample group clusters. PC: phenolic compound clusters; PA: phenolic acids; FL: flavonoids. Avocado samples mentioned in abbreviations are: REPEL (Reed peel); REPUL (Reed pulp); RES (Reed seed); RHPEL (ripe Hass peel); RHPUL (ripe Hass pulp); RHS (ripe Hass seed); URHPEL (unripe Hass peel); URHPUL (unripe Hass pulp); URHS (unripe Hass seed); WZPEL (Wurtz peel); WZPUL (Wurtz pulp) and WZS (Wurtz seed).

A hierarchical heat map was constructed for further analysing of HPLC-PDA data of 10 phenolic compounds (five phenolic acids and five flavonoids) of selected samples, as shown in Figure 3. Four clusters are generated in rows and columns and shown in a hierarchical cluster.

The difference in clustering and colour in the heatmap showed the difference in the concentration of phenolic compounds. Reed pulp contains a higher concentration of epicatechin, protocatechuic acid, and kaempferol. WZPEL contain a higher concentration of hydroxybenzoic acid. Similarly, chlorogenic acid and catechin are found in AG-4 group. Epicatechin was reported to exist in high quantities in avocado pulps [64], which may explain the high concentration of epicatechin observed in Reed pulp sample’s HPLC analysis. Previous studies showed that chlorogenic acid was predominantly found in the peel as compared to the pulp [46]; however, our results showed that all pulp samples had relatively high quantities of chlorogenic acid. Ripe Hass seed appeared to be the sample with the least concentration of the 10 phenolic compounds. Unripe was categorised under the same avocado group (AG-4), whereas Reeds and Wurtz saw none of the part samples placed in the same avocado group. Overall, except for unripe Hass, all the samples contained relatively different phenolic profiles, each offering unique propositions for commercial purposes. Previously, it has been mentioned that the degree of ripeness of the avocado fruit may cause changes in its bioactive compound levels, with a general increase in total phenol content observed but a slight decrease in flavonoids [65].

## 4. Conclusions

In conclusion, several novel findings were draw from this study which were not previously described in the literature. Ten different assays were performed to remark the amount phenolic content and antioxidant capacity of selected three different Australian grown avocado. The finding on Avocado by-products extended new limelight and changed the thought of importance for human diet. Peel and seed contain a remarkable source of polyphenols. The identification of 134 compounds was enabled by applying an advanced and comprehensive tool, LC-ESI-QTOF-MS/MS. Quantification of HPLC-PDA showed that epicatechin, protocatechuic acid, and kaempferol (>1 mg/g) has a higher concentration in reed pulp. Unripe Hass pulp is rich in flavonoids and phenolic acids. Avocado peel shows the higher phenolic content and antioxidant capacity than seed and pulp. The TPC and TTC has higher correlation with antioxidant activity. The study supported that avocado peel and seed are a potential food-waste source of polyphenol, higher antioxidant capacity that could be used in feed, functional food, nutraceuticals, and cosmetics. In the future, bioaccessibility, bioavailabity and toxicology, and animal models are required for commercialization of Avocado waste.

## Figures and Tables

**Table 1:** Polyphenol content and antioxidant activity detected in different fresh avocado peels, seeds, and pulps.

Assays	Avocado Peel				Avocado Seed				Avocado Pulp			
	Hass (Ripe)	Hass (Unripe)	Reed	Wurtz	Hass (Ripe)	Hass (Unripe)	Reed	Wurtz	Hass (Ripe)	Hass (Unripe)	Reed	Wurtz
TPC (mg GAE/g)	77.85 ± 3.20 ^a^	45.74 ± 2.08 ^b^	29.22 ± 0.47 ^c^	49.18 ± 2.23 ^b^	36.82 ± 2.58 ^b^	26.93 ± 2.21 ^c^	36.20 ± 0.52 ^b^	44.91 ± 4.44 ^a^	0.26 ± 0.02 ^ab^	0.20 ± 0.01 ^b^	0.28 ± 0.01 ^a^	0.25 ± 0.05 ^ab^
TFC (mg QE/g)	1.06 ± 0.06 ^b^	3.44 ± 0.03 ^a^	0.38 ± 0.01 ^d^	0.91 ± 0.04 ^c^	0.39 ± 0.01 ^b^	2.75 ± 0.24 ^a^	0.06 ± 0.01 ^c^	0.26 ± 0.01 ^b^	0.09 ± 0.01 ^a^	0.04 ± 0.01 ^b^	0.02 ± 0.01 ^c^	0.01 ± 0.01 ^c^
TTC (mg CE/g)	148.98 ± 9.20 ^a^	85.84 ± 2.70 ^b^	29.34 ± 2.57 ^d^	53.60 ± 0.72 ^c^	58.26 ± 4.30 ^a^	40.85 ± 1.16 ^c^	42.94 ± 1.10 ^c^	51.73 ± 2.09 ^b^	-	-	-	-
DPPH (mg AAE/g)	71.03 ± 3.05 ^a^	57.82 ± 1.22 ^c^	41.53 ± 0.25 ^d^	66.13 ± 2.34 ^b^	47.97 ± 3.96 ^b^	39.36 ± 1.40 ^c^	49.97 ± 2.34 ^b^	56.00 ± 1.84 ^a^	0.10 ± 0.01 ^c^	0.13 ± 0.01 ^b^	0.08 ± 0.01 ^d^	0.16 ± 0.01 ^a^
FRAP (mg AAE/g)	3.05 ± 0.27 ^a^	1.00 ± 0.06 ^b^	0.19 ± 0.01 ^c^	0.20 ± 0.01 ^c^	0.98 ± 0.08 ^c^	0.87 ± 0.07 ^c^	1.29 ± 0.05 ^b^	3.69 ± 0.10 ^a^	0.08 ± 0.01 ^a^	0.06 ± 0.01 ^b^	0.06 ± 0.01 ^b^	0.02 ± 0.01 ^c^
ABTS (mg AAE/g)	75.77 ± 2.47 ^a^	39.05 ± 1.05 ^c^	38.30 ± 1.99 ^c^	66.04 ± 4.44 ^b^	74.14 ± 2.66 ^a^	28.29 ± 2.62 ^c^	27.42 ± 0.40 ^c^	55.87 ± 3.17 ^b^	0.40 ± 0.04 ^a^	0.40 ± 0.02 ^a^	0.34 ± 0.02 ^b^	0.35 ± 0.03 ^b^
RPA (mg AAE/g)	24.45 ± 1.21 ^a^	11.32 ± 1.43 ^c^	14.78 ± 2.12 ^b^	9.37 ± 2.94 ^d^	13.07 ± 2.31 ^a^	7.35 ± 0.29 ^b^	5.52 ± 1.31 ^b^	14.28 ± 3.12 ^a^	1.47 ± 0.09 ^a^	0.91 ± 0.12 ^c^	0.17 ± 0.09 ^d^	0.97 ± 0.03 ^b^
·OH-RSA (mg AAE/g)	7.29 ± 0.07 ^c^	9.75 ± 0.31 ^a^	8.14 ± 0.12 ^b^	3.68 ± 0.47 ^d^	4.24 ± 0.12 ^c^	1.47 ± 0.09 ^d^	13.25 ± 0.41 ^a^	7.48 ± 0.09 ^b^	0.78 ± 0.04 ^b^	1.14 ± 0.11 ^a^	0.34 ± 0.13 ^c^	0.14 ± 0.04 ^d^
FICA (mg EDTA/g)	4.12 ± 0.38 ^a^	2.41 ± 0.14 ^b^	2.17 ± 0.04 ^b^	1.91 ± 0.24 ^c^	5.39 ± 0.12 ^b^	3.14 ± 0.09 ^c^	9.68 ± 0.12 ^a^	1.97 ± 0.21 ^d^	0.17 ± 0.09 ^c^	0.47 ± 0.01 ^b^	1.02 ± 0.04 ^a^	0.42 ± 0.13 ^b^
TAC (mg AAE/g)	34.05 ± 0.96 ^a^	9.25 ± 0.22 ^b^	11.85 ± 0.34 ^b^	35.02 ± 1.27 ^a^	27.49 ± 1.04 ^a^	13.26 ± 0.28 ^c^	6.58 ± 0.25 ^d^	19.48 ± 0.35 ^b^	0.26 ± 0.01 ^b^	0.25 ± 0.01 ^b^	0.31 ± 0.02 ^a^	0.33 ± 0.01 ^a^

Values represented as mean ± standard deviation obtained from seven measurements; Values with different letters (^a–d^) along the row indicate significant statistical differences (95% significance).

**Table 2:** Pearson’s correlation coefficient (r) for the relationships between assays for antioxidant capacity and phenolic contents.

Variables	TPC	TFC	TTC	DPPH	FRAP	ABTS	RPA	·OH-RSA	FICA
TFC	0.403								
TTC	0.948 **	0.478							
DPPH	0.964 **	0.449	0.851 **						
FRAP	0.714 **	0.113	0.690 **	0.631 *					
ABTS	0.907 **	0.253	0.814 **	0.910 **	0.609 *				
RPA	0.909 **	0.294	0.891 **	0.839 **	0.711 **	0.878 **			
·OH-RSA	0.657 *	0.204	0.560	0.709 **	0.506	0.482	0.553		
FICA	0.490	0.040	0.423	0.543	0.338	0.423	0.323	0.722 **	
TAC	0.853 **	0.201	0.760 **	0.836 **	0.512	0.954 **	0.797 **	0.279	0.297

* Represents significant correlation at *p* < 0.05; ** represents a highly significant correlation at *p* < 0.01.

**Table 3:** Characterization of phenolic compounds in different avocado samples by LC-ESI-QTOF-MS/MS.

No.	Molecular Formula	Proposed Compounds	RT (min)	Ionization (ESI^+^/ESI^−^)	Molecular Weight	Theoretical (*m*/*z*)	Observed (*m*/*z*)	Error (ppm)	MS/MS Production	Avocado
**Phenolic acid**										
**Hydroxybenzoic acids**										
1	C_14_H_6_O_8_	Ellagic acid	5.872	[M-H]^−^	302.0063	300.9990	301.0004	4.7	284, 229, 201	* URHPUL, WZPUL
2	C_7_H_6_O_4_	2,3-Dihydroxybenzoic acid	11.036	** [M-H]^−^	154.0266	153.0193	153.0195	1.3	109	* RES, RHS, URHS
3	C_13_H_16_O_9_	Protocatechuic acid 4-*O*-glucoside	11.086	** [M-H]^−^	316.0794	315.0721	315.0718	−1.0	153	* RES, REPEL, RHPEL, RHPUL, URHS, WZPEL
4	C_13_H_16_O_8_	4-Hydroxybenzoic acid 4-*O*-glucoside	11.103	** [M-H]^−^	300.0845	299.0772	299.0766	−2.0	255, 137	* RES, URHPEL, URHS
5	C_8_H_8_O_5_	4-*O*-Methylgallic acid	12.847	[M+H]^+^	184.0372	185.0445	185.0447	1.1	170, 142	* WZS, REPEL, RES, RHPEL, WZPEL
6	C_13_H_16_O_10_	Galloyl glucose	12.908	** [M-H]^−^	332.0743	331.0670	331.0682	3.6	169, 125	* RES, RHPEL, RHPUL, WZPUL
7	C_7_H_6_O_5_	Gallic acid	12.958	** [M-H]^−^	170.0215	169.0142	169.0136	−3.6	125	* RES, REPEL, URHPEL, URHS
8	C_9_H_10_O_5_	3,4-*O*-Dimethylgallic acid	19.870	** [M+H]^+^	198.0528	199.0601	199.0598	−1.5	153, 139, 125, 111	* WZS, REPEL, RHPEL, RHS, URHPEL, URHS, WZPEL
9	C_7_H_6_O_3_	2-Hydroxybenzoic acid	21.117	** [M-H]^−^	138.0317	137.0244	137.0245	0.7	93	* WZS, RES, RHPEL, RHPUL, RHS, URHS, WZPUL
10	C_23_H_28_O_11_	Paeoniflorin	40.792	** [M-H]^−^	480.1632	479.1559	479.1556	−0.6	449, 357, 327	* RHPEL, REPEL, RES, URHPEL, WZPEL
**Hydroxycinnamic acids**										
11	C_16_H_20_O_9_	Ferulic acid 4-*O*-glucoside	4.198	** [M-H]^−^	356.1107	355.1034	355.1026	−2.3	193, 178, 149,134	* WZPEL, REPEL, URHPEL, WZS
12	C_33_H_40_O_18_	1-Sinapoyl-2-feruloylgentiobiose	4.455	** [M-H]^−^	724.2215	723.2142	723.2164	3.0	529, 499	* WZPUL, RHPUL, URHPEL, URHPUL
13	C_9_H_8_O_5_	Hydroxycaffeic acid	5.288	[M-H]^−^	196.0372	195.0299	195.0295	−2.1	151	* RES
14	C_43_H_48_O_21_	1-Sinapoyl-2,2′-diferuloylgentiobiose	7.114	[M-H]^−^	900.2688	899.2615	899.2643	3.1	613, 201	* REPUL
15	C_9_H_8_O_2_	Cinnamic acid	12.544	** [M-H]^−^	148.0524	147.0451	147.0458	4.8	103	* RES, REPEL, RHPUL, RHS, URHPEL, URHPUL, WZPEL, WZPUL
16	C_13_H_12_O_8_	*p*-Coumaroyl tartaric acid	14.300	** [M-H]^−^	296.0532	295.0459	295.0446	−4.4	115	* RES, REPEL, RHPEL, URHPEL, WZPEL, WZS
17	C_16_H_18_O_9_	3-Caffeoylquinic acid	16.837	** [M-H]^−^	354.0951	353.0878	353.0875	−0.8	253, 190, 144	* URHS, REPEL, RES, RHPEL, RHS, URHPEL, URHPUL, WZPEL, WZS
18	C_9_H_8_O_4_	Caffeic acid	16.854	** [M-H]^−^	180.0423	179.0350	179.0351	0.6	143, 133	* URHS, RHPEL, URHPEL
19	C_18_H_22_O_10_	3-Sinapoylquinic acid	17.799	** [M-H]^−^	398.1213	397.1140	397.1155	3.8	233, 179	* WZPEL, REPEL, RHPEL, RHPUL, RHS, URHPEL, WZPUL
20	C_15_H_18_O_9_	Caffeoyl glucose	18.676	[M-H]^−^	342.0951	341.0878	341.0882	1.2	179, 161	* RHPEL, WZPUL
21	C_29_H_36_O_15_	Verbascoside	19.887	** [M-H]^−^	624.2054	623.1981	623.1976	−0.8	477, 461, 315, 135	* REPUL, URHS
22	C_15_H_16_O_10_	Caffeic acid 3-*O*-glucuronide	21.989	** [M-H]^−^	356.0743	355.0670	355.0662	−2.3	179	* RHPEL, RES, RHS, URHS
23	C_16_H_18_O_8_	3-*p*-Coumaroylquinic acid	22.205	** [M-H]^−^	338.1002	337.0929	337.0911	−5.3	265, 173, 162	* URHS, REPEL, REPUL, RES, RHS, URHPEL, URHPUL, WZPUL, WZS, WZPEL
24	C_10_H_10_O_4_	Isoferulic acid	23.304	** [M-H]^−^	194.0579	193.0506	193.0502	−2.1	178, 149, 134	* WZS, REPEL, RES, RHPUL, RHS, URHPEL, URHS, WZPEL, WZPUL
25	C_18_H_17_NO_5_	*p*-Coumaroyl tyrosine	25.151	[M-H]^−^	327.1107	326.1034	326.1020	−4.3	282	* RES, WZPEL
26	C_17_H_20_O_9_	3-Feruloylquinic acid	25.259	** [M-H]^−^	368.1107	367.1034	367.1026	−2.2	298, 288, 192, 191	* WZS, REPEL, RES, RHPEL, RHS, URHS, WZPEL
27	C_15_H_18_O_8_	*p*-Coumaric acid 4-*O*-glucoside	25.347	[M-H]^−^	326.1002	325.0929	325.0941	3.7	163	* REPEL
28	C_11_H_12_O_5_	Sinapic acid	26.021	** [M-H]^−^	224.0685	223.0612	223.0613	0.4	205, 163	* WZS, REPEL, REPUL, RES, RHPEL, RHPUL, RHS, URHPEL, URHS, WZPEL, WZPUL
29	C_9_H_8_O_3_	*m*-Coumaric acid	31.217	** [M-H]^−^	164.0473	163.0400	163.0403	1.8	119	* RHPEL, REPEL, REPUL, RES, RHS, URHPEL, URHS, WZPEL, WZPUL, WZS
30	C_18_H_16_O_8_	Rosmarinic acid	32.802	** [M-H]^−^	360.0845	359.0772	359.0787	4.2	179	* REPEL, RHS, URHPEL, URHS, WZS
31	C_25_H_24_O_12_	1,5-Dicaffeoylquinic acid	50.465	** [M-H]^−^	516.1268	515.1195	515.1212	3.3	353, 335, 191, 179	* RES, REPEL, RHPEL, WZPEL
**Hydroxyphenylacetic acids**										
32	C_8_H_8_O_4_	3,4-Dihydroxyphenylacetic acid	14.004	** [M-H]^−^	168.0423	167.0350	167.0353	1.8	149, 123	* URHS, REPEL, RES, RHPEL, RHS, URHPEL, WZPEL, WZS
33	C_8_H_8_O_3_	2-Hydroxy-2-phenylacetic acid	24.027	** [M-H]^−^	152.0473	151.0400	151.0396	−2.6	136, 92	* URHS, REPUL, RES, RHPEL, RHPUL, RHS, URHPUL, WZPUL, WZS
**Hydroxyphenylpropanoic acids**										
34	C_10_H_12_O_7_S	Dihydroferulic acid 4-sulfate	4.082	** [M-H]^−^	276.0304	275.0231	275.0225	−2.2	195, 177, 151	* WZPEL, REPEL, WZS
35	C_16_H_20_O_10_	Dihydroferulic acid 4-*O*-glucuronide	16.479	[M-H]^−^	372.1056	371.0983	371.0991	2.2	195	* WZS, RES, RHS, URHS
36	C_9_H_10_O_4_	3-Hydroxy-3-(3-hydroxyphenyl)propionic acid	31.233	[M-H]^−^	182.0579	181.0506	181.0512	3.3	163, 135, 119	* RHPEL
**Flavonoid**										
**Anthocyanins**										
37	C_27_H_31_O_14_	Pelargonidin 3-*O*-rutinoside	10.679	[M+H]^+^	579.1714	580.1787	580.1794	1.2	433, 271	* WZPUL
38	C_27_H_31_O_17_	Delphinidin 3-*O*-glucosyl-glucoside	37.230	** [M+H]^+^	627.1561	628.1634	628.1619	−2.4	465, 303	* WZPEL, REPEL, RHPEL, RHS, URHPEL, URHS, WZS,
39	C_28_H_33_O_17_	Petunidin 3,5-*O*-diglucoside	40.846	[M+H]^+^	641.1718	642.1791	642.1794	0.5	479, 317	* URHPEL, RHS
40	C_27_H_31_O_16_	Cyanidin 3,5-*O*-diglucoside	42.367	** [M+H]^+^	611.1612	612.1685	612.1664	−3.4	449, 287	* REPEL, RHPEL, URHPEL, WZPEL,
41	C_21_H_21_O_12_	Delphinidin 3-*O*-glucoside	45.306	** [M+H]^+^	465.1033	466.1106	466.1098	−1.7	303	* RES, REPEL, RHPEL, RHS, URHPEL, URHS, WZPEL, WZS
42	C_21_H_21_O_11_	Cyanidin 3-*O*-galactoside	48.907	** [M+H]^+^	449.1084	450.1157	450.1143	−3.1	287	* WZPEL, REPEL, RES, URHPEL, URHS, WZS
43	C_21_H_21_O_10_	Isopeonidin 3-*O*-arabinoside	52.693	[M+H]^+^	433.1135	434.1208	434.1200	−1.8	271, 253, 243	* RES, REPUL
44	C_24_H_25_O_13_	Petunidin 3-*O*-(6″-acetyl-glucoside)	61.318	[M+H]^+^	521.1295	522.1368	522.1372	0.8	317	* URHPEL
45	C_30_H_27_O_13_	Cyanidin 3-*O*-(6″-p-coumaroyl-glucoside)	84.651	** [M+H]^+^	595.1452	596.1525	596.1510	−2.5	287	* WZPEL
**Dihydrochalcones**										
46	C_21_H_24_O_11_	3-Hydroxyphloretin 2′-*O*-glucoside	19.046	** [M-H]^−^	452.1319	451.1246	451.1249	0.7	289, 273	* WZS, REPEL, REPUL, RES, RHPEL, RHPUL, RHS, URHPEL, URHS, WZPEL, WZPUL
47	C_21_H_24_O_10_	Phloridzin	46.862	** [M-H]^−^	436.1369	435.1296	435.1308	2.8	273	* WZS, REPEL, RES, RHPEL, RHPUL, RHS, URHPEL, URHS
**Dihydroflavonols**										
48	C_15_H_12_O_7_	Dihydroquercetin	26.462	** [M-H]^−^	304.0583	303.0510	303.0502	−2.6	285, 275, 151	* URHS, REPUL, RES, RHPEL, RHS, URHPEL, WZPEL, WZS
49	C_21_H_22_O_12_	Dihydromyricetin 3-*O*-rhamnoside	35.541	** [M-H]^−^	466.1111	465.1038	465.1035	−0.6	301	* URHS, REPEL, REPUL, RES, RHPEL, RHS, URHPEL, WZPEL, WZPUL, WZS
50	C_21_H_22_O_11_	Dihydroquercetin 3-*O*-rhamnoside	53.449	** [M-H]^−^	450.1162	449.1089	449.1095	1.3	303	* URHS, RES, RHPEL, WZS
**Flavanols**										
51	C_30_H_26_O_14_	Prodelphinidin dimer B3	15.427	** [M+H]^+^	610.1323	611.1396	611.1409	2.1	469, 311, 291	* RHPEL, REPEL, RHS, URHPEL, WZPEL
52	C_22_H_18_O_10_	(+)-Catechin 3-*O*-gallate	22.318	** [M-H]^−^	442.0900	441.0827	441.0840	2.9	289, 169, 125	* RES, REPEL, RHPEL
53	C_15_H_14_O_7_	(-)-Epigallocatechin	25.027	** [M-H]^−^	306.0740	305.0667	305.0674	2.3	261, 219	* WZS, REPEL, URHPEL, URHS, WZPEL
54	C_30_H_26_O_12_	Procyanidin dimer B1	26.192	** [M-H]^−^	578.1424	577.1351	577.1368	2.9	451	* REPEL, RES, RHPEL, RHS, URHPEL, URHS, WZPEL, WZS
55	C_60_H_50_O_24_	Cinnamtannin A2	29.030	** [M-H]^−^	1154.2692	1153.2619	1153.2673	4.7	739	* RHPEL, REPEL, RES, RHS, URHPEL, URHS, WZPEL, WZS
56	C_22_H_18_O_11_	(+)-Gallocatechin 3-*O*-gallate	29.655	[M-H]^−^	458.0849	457.0776	457.0777	0.2	305, 169	* REPEL, RHS, URHPEL
57	C_15_H_14_O_6_	(-)-Epicatechin	31.233	** [M-H]^−^	290.0790	289.0717	289.0728	3.8	245, 205, 179	* URHS, REPEL, RES, RHPEL, URHPEL, URHPUL, WZPEL, WZPUL, WZS
58	C_45_H_38_O_18_	Procyanidin trimer C1	33.608	** [M-H]^−^	866.2058	865.1985	865.2010	2.9	739, 713, 695	* WZS, REPEL, RES, RHPEL, RHS, URHPEL, URHS, WZPEL
59	C_16_H_16_O_6_	3′-*O*-Methylcatechin	43.161	** [M-H]^−^	304.0947	303.0874	303.0879	1.6	271, 163	* RHPEL, REPEL, RHS
60	C_22_H_24_O_13_	4′-*O*-Methyl-(-)-epigallocatechin 7-*O*-glucuronide	58.945	** [M-H]^−^	496.1217	495.1144	495.1161	3.4	451, 313	* REPEL, RHPEL, RHPUL, URHS, WZPUL, WZS,
**Flavanones**										
61	C_27_H_32_O_14_	Naringin	35.911	** [M-H]^−^	580.1792	579.1719	579.1736	2.9	271	* WZS, URHPEL, WZPEL
62	C_28_H_30_O_18_	Hesperetin 3′,7-*O*-diglucuronide	42.184	** [M-H]^−^	654.1432	653.1359	653.1369	1.5	477, 301, 286, 242	* RHPEL, REPUL
63	C_20_H_20_O_5_	8-Prenylnaringenin	45.759	[M+H]^+^	340.1311	341.1384	341.1383	−0.3	323, 137	* WZS, REPEL, URHPEL, URHS, WZPEL
64	C_28_H_34_O_15_	Hesperidin	50.645	[M+H]^+^	610.1898	611.1971	611.1987	2.6	593, 465, 449, 303	* WZS
65	C_22_H_22_O_12_	Hesperetin 3′-*O*-glucuronide	52.488	** [M-H]^−^	478.1111	477.1038	477.1045	1.5	301, 175, 113, 85	* URHS, REPEL, RHPEL, WZPEL
66	C_27_H_32_O_15_	Eriocitrin	54.531	** [M-H]^−^	596.1741	595.1668	595.1656	−2.0	431, 287	* URHPEL, REPEL, WZPEL
**Flavones**										
67	C_15_H_10_O_4_	7,4′-Dihydroxyflavone	18.251	[M+H]^+^	254.0579	255.0652	255.0643	−3.5	227, 199, 171	* REPEL
68	C_28_H_32_O_15_	Neodiosmin	32.723	[M+H]^+^	608.1741	609.1814	609.1812	−0.3	301, 286	* WZS
69	C_19_H_18_O_7_	Gardenin B	41.653	** [M+H]^+^	358.1053	359.1126	359.1120	−1.7	344, 329, 311	* WZPUL, REPEL, RHPEL
70	C_21_H_20_O_10_	Apigenin 6-C-glucoside	52.809	** [M-H]^−^	432.1056	431.0983	431.0974	−2.1	413, 341, 311	* WZS, REPEL, RHS, URHS, WZPEL
71	C_22_H_22_O_11_	Chrysoeriol 7-*O*-glucoside	54.226	** [M+H]^+^	462.1162	463.1235	463.1255	4.3	445, 427, 409, 381	* RHPEL, REPEL, RES, RHS, URHS, WZPEL,
72	C_27_H_30_O_15_	Apigenin 6,8-di-C-glucoside	56.081	** [M-H]^−^	594.1585	593.1512	593.1516	0.7	503, 473	* RES, URHPEL, URHS, WZPEL
73	C_21_H_20_O_11_	6-Hydroxyluteolin 7-*O*-rhamnoside	57.771	** [M-H]^−^	448.1006	447.0933	447.0934	0.2	301	* RES, URHS, REPEL, RHPEL, RHS, WZPEL, WZS
74	C_26_H_28_O_14_	Apigenin 7-*O*-apiosyl-glucoside	59.215	[M+H]^+^	564.1479	565.1552	565.1542	−1.8	296	* URHPEL
75	C_18_H_16_O_7_	Cirsilineol	69.389	** [M+H]^+^	344.0896	345.0969	345.0958	−3.2	330, 312, 297, 284	* RES
**Flavonols**										
76	C_26_H_26_O_17_	Quercetin 3-*O*-xylosyl-glucuronide	15.319	** [M+H]^+^	610.1170	611.1243	611.1224	−3.1	479, 303, 285, 239	* REPEL, URHS
77	C_32_H_38_O_20_	Quercetin 3-*O*-xylosyl-rutinoside	18.863	** [M+H]^+^	742.1956	743.2029	743.2023	−0.8	479, 317	* REPEL, URHPEL, URHS, WZPEL
78	C_22_H_24_O_9_	3-Methoxynobiletin	20.837	** [M+H]^+^	432.1420	433.1493	433.1482	−2.5	403, 385, 373, 345	* URHPEL, RES, WZPEL
79	C_21_H_22_O_8_	3-Methoxysinensetin	23.577	** [M+H]^+^	402.1315	403.1388	403.1402	3.5	388, 373, 355, 327	* URHPUL, REPEL, RES, URHS
80	C_20_H_18_O_12_	Myricetin 3-*O*-arabinoside	24.524	** [M-H]^−^	450.0798	449.0725	449.0728	0.7	317	* RHPEL, RHPUL, RHS, URHPUL, WZS,
81	C_33_H_40_O_20_	Kaempferol 3-*O*-glucosyl-rhamnosyl-galactoside	24.867	** [M-H]^−^	756.2113	755.2040	755.2068	3.7	285	* REPEL, WZPEL
82	C_30_H_32_O_20_	Quercetin 3-*O*-(6”-malonyl-glucoside) 7-*O*-glucoside	31.133	[M+H]^+^	712.1487	713.1560	713.1547	−1.8	551, 303	* REPUL, RES, RHS, URHPEL, WZS
83	C_27_H_30_O_17_	Myricetin 3-*O*-rutinoside	34.005	** [M-H]^−^	626.1483	625.1410	625.1423	2.1	301	* URHPEL, REPEL, RES, RHPEL, RHS, URHS, WZPEL, WZS
84	C_27_H_30_O_16_	Kaempferol 3,7-*O*-diglucoside	40.146	** [M-H]^−^	610.1534	609.1461	609.1457	−0.7	447, 285	* RHPEL, RES, URHPEL, URHS, WZS
85	C_26_H_28_O_16_	Quercetin 3-*O*-glucosyl-xyloside	41.207	** [M-H]^−^	596.1377	595.1304	595.1296	−1.3	265, 138, 116	* RHPEL, RES, RHS, URHPEL
86	C_15_H_10_O_10_S	Quercetin 3′-sulfate	41.985	[M-H]^−^	381.9995	380.9922	380.9937	3.9	301	* RHPEL
87	C_21_H_18_O_13_	Quercetin 3′-*O*-glucuronide	44.818	** [M-H]^−^	478.0747	477.0674	477.0695	4.4	301	* RHPEL, URHPEL
88	C_26_H_28_O_15_	Kaempferol 3-*O*-xylosyl-glucoside	45.009	** [M+H]^+^	580.1428	581.1501	581.1480	−3.6	419, 401, 383	* RHPEL, URHS, URHPEL, WZPEL, WZS
89	C_16_H_12_O_7_	Isorhamnetin	50.120	** [M-H]^−^	316.0583	315.0510	315.0514	1.3	300, 271	* RHPEL, URHPEL, WZPEL, REPEL
90	C_21_H_20_O_12_	Myricetin 3-*O*-rhamnoside	53.449	** [M-H]^−^	464.0955	463.0882	463.0886	0.9	317	* URHS, RES, RHPEL, RHPUL, RHS, URHPEL, WZPUL, WZS,
91	C_24_H_22_O_15_	Quercetin 3-O-(6″-malonyl-glucoside)	54.576	** [M+H]^+^	534.1010	533.0937	533.0916	−3.9	303	* RHPEL, REPEL, REPUL, RES
92	C_33_H_40_O_19_	Kaempferol 3-*O*-(2″-rhamnosyl-galactoside) 7-*O*-rhamnoside	59.352	** [M-H]^−^	740.2164	739.2091	739.2089	−0.3	593, 447, 285	* URHPEL, REPEL, RHPEL, WZPEL
**Isoflavonoids**										
93	C_17_H_16_O_5_	Sativanone	12.359	** [M-H]^−^	300.0998	299.0925	299.0928	1.0	284, 269, 225	* RHS, URHPEL, URHS
94	C_18_H_18_O_6_	3′-*O*-Methylviolanone	14.768	[M-H]^−^	330.1103	329.1030	329.1019	−3.3	314, 299, 284, 256	* REPUL
95	C_16_H_14_O_5_	Dihydrobiochanin A	15.236	[M+H]^+^	286.0841	287.0914	287.0911	−1.0	269, 203, 201, 175	* REPEL
96	C_24_H_22_O_12_	6″-*O*-Malonyldaidzin	16.246	[M+H]^+^	502.1111	503.1184	503.1200	3.2	255	* REPEL
97	C_17_H_16_O_6_	Violanone	26.247	** [M-H]^−^	316.0947	315.0874	315.0866	−2.5	300, 285, 135	* RHPEL, REPEL, REPUL, RES, RHPEL, URHPEL, URHPUL, WZPEL
98	C_17_H_14_O_6_	2′,7-Dihydroxy-4′,5′-dimethoxyisoflavone	29.218	** [M+H]^+^	314.0790	315.0863	315.0868	1.6	300, 282	* URHPEL, RHS, URHS, WZPEL
99	C_15_H_12_O_5_	3′,4′,7-Trihydroxyisoflavanone	31.267	** [M-H]^−^	272.0685	271.0612	271.0616	1.5	177, 151, 119, 107	* URHS, REPEL, RES, RHPEL, RHS, URHPEL, URHPUL, WZPEL, WZS
100	C_15_H_10_O_5_	3′-Hydroxydaidzein	31.654	** [M+H]^+^	270.0528	271.0601	271.0612	4.1	253, 241, 225	* RHS, REPUL, RHPEL, URHS, WZPEL, WZS
101	C_15_H_10_O_6_	3′-Hydroxygenistein	32.748	** [M+H]^+^	286.0477	287.0550	287.0557	2.4	269, 259	* RHS, REPEL, RES, RHPEL, URHPEL, URHS, WZPEL, WZS
102	C_16_H_12_O_5_	2′-Hydroxyformononetin	37.823	[M+H]^+^	284.0685	285.0758	285.0749	−3.2	270, 229	* RHPUL, RES
103	C_15_H_10_O_7_	5,6,7,3′,4′-Pentahydroxyisoflavone	44.414	** [M+H]^+^	302.0427	303.0500	303.0505	1.6	285, 257	* URHS, REPEL, RES, RHPEL, RHS, URHPEL, WZPEL, WZS
104	C_23_H_22_O_10_	6″-*O*-Acetyldaidzin	46.922	** [M-H]^−^	458.1213	457.1140	457.1163	5.0	221	* RHPEL, WZPEL
105	C_24_H_22_O_13_	6″-*O*-Malonylgenistin	64.036	[M+H]^+^	518.1060	519.1133	519.1134	0.2	271	* RHS, REPEL, URHPEL
106	C_15_H_12_O_4_	2-Dehydro-*O*-desmethylangolensin	75.663	[M-H]^−^	256.0736	255.0663	255.0671	3.1	135, 119	* RES
**Lignans**										
107	C_23_H_28_O_6_	Schisandrin B	7.433	** [M+H]^+^	400.1886	401.1959	401.1956	−0.7	386	* WZPEL, RES
108	C_20_H_18_O_6_	Episesamin	7.775	[M-H]^−^	354.1103	353.1030	353.1020	−2.8	338, 163	* URHS
109	C_20_H_24_O_7_	Todolactol A	13.426	[M-H]^−^	376.1522	375.1449	375.1467	4.8	313, 137	* REPUL
110	C_20_H_22_O_7_	7-Hydroxymatairesinol	14.834	[M-H]^−^	374.1366	373.1293	373.1291	−0.5	343, 313, 298, 285	* URHPUL, REPEL, REPUL, WZPUL
111	C_21_H_24_O_6_	Arctigenin	29.065	** [M-H]^−^	372.1573	371.1500	371.1509	2.4	356, 312, 295	* URHPUL, REPEL, RES, RHS, URHS, WZPEL, WZPUL, WZS
112	C_20_H_20_O_7_	7-Oxomatairesinol	32.723	** [M+H]^+^	372.1209	373.1282	373.1275	−1.9	358, 343, 328, 325	* REPUL, RES, RHPUL, URHPUL, WZPUL
113	C_20_H_22_O_6_	Matairesinol	45.926	** [M-H]^−^	358.1416	357.1343	357.1348	1.4	342, 327, 313, 221	* RES, REPEL, URHPEL, URHS, WZPEL, WZS
114	C_22_H_24_O_6_	Schisandrin C	59.344	** [M+H]^+^	384.1573	385.1646	385.1663	4.4	370, 315, 300	* REPEL, URHPEL, URHS, WZPEL, WZPUL
115	C_30_H_38_O_10_	Secoisolariciresinol-sesquilignan	59.607	[M-H]^−^	558.2465	557.2392	557.2387	−0.9	539, 521, 509, 361	* REPEL, RHPEL
116	C_23_H_28_O_7_	Schisandrol B	63.253	[M+H]^+^	416.1835	417.1908	417.1929	5.0	224, 193, 165	* REPEL
117	C_20_H_20_O_6_	Conidendrin	76.546	** [M+H]^+^	356.1260	357.1333	357.1328	−1.4	339, 221, 206	* RHPEL, RHS, URHPEL, WZPEL
**Stilbenes**										
118	C_14_H_12_O_3_	Resveratrol	31.283	** [M-H]^−^	228.0786	227.0713	227.0724	4.8	212, 185, 157, 143	* URHS, REPEL, RES, RHPEL, RHS, WZPEL, WZS
119	C_17_H_18_O_4_	4′-Hydroxy-3,4,5-trimethoxystilbene	63.229	[M+H]^+^	286.1205	287.1278	287.1273	−1.7	271, 241, 225	* RHPEL, REPUL, RHPUL, URHPEL, URHPUL, URHS, WZPEL, WZS
**Other polyphenols**										
**Alkylmethoxyphenols**										
120	C_15_H_14_O_3_	4-Vinylsyringol	12.295	[M+H]^+^	242.0943	243.1016	243.1017	0.4	255, 211, 197	* RES
**Furanocoumarins**										
121	C_13_H_10_O_5_	Isopimpinellin	27.861	[M+H]^+^	246.0528	247.0601	247.0607	2.4	232, 217, 205, 203	* RHS, REPEL, RHPEL, URHPEL, URHS, WZPEL
**Hydroxybenzaldehydes**										
122	C_8_H_8_O_2_	*p*-Anisaldehyde	17.690	** [M+H]^+^	136.0524	137.0597	137.0598	0.7	122, 109	* URHPEL, REPEL, RES, RHPEL, RHS, URHPUL, URHS, WZPEL, WZS
**Hydroxybenzoketones**										
123	C_9_H_10_O_7_S	2-Hydroxy-4-methoxyacetophenone 5-sulfate	12.908	** [M-H]^−^	262.0147	261.0074	261.0084	3.8	181, 97	* RES, RHPUL, WZPEL
**Hydroxycoumarins**										
124	C_15_H_16_O_9_	Esculin	17.940	** [M+H]^+^	340.0794	341.0867	341.0860	−2.1	179, 151	* RHS, URHS, WZS
125	C_9_H_6_O_2_	Coumarin	22.283	** [M+H]^+^	146.0368	147.0441	147.0448	4.8	103, 91	* RES, REPEL, RHPUL, RHS, URHPEL
126	C_9_H_6_O_4_	Esculetin	24.542	[M-H]^−^	178.0266	177.0193	177.0201	4.5	149, 133, 89	* WZPEL
127	C_10_H_8_O_4_	Scopoletin	31.863	** [M-H]^−^	192.0423	191.0350	191.0358	4.2	176	* URHS, RHPEL, URHPEL, WZPEL, WZPUL, WZS
**Hydroxyphenylpropenes**										
128	C_10_H_12_O_2_	2-Methoxy-5-prop-1-enylphenol	25.818	[M+H]^+^	164.0837	165.0910	165.0903	−4.2	149, 137, 133, 124	* WZPEL, REPEL, URHPEL
**Other polyphenols**										
129	C_26_H_20_O_10_	Salvianolic acid C	35.209	** [M-H]^−^	492.1056	491.0983	491.0987	0.8	311, 267, 249	* URHS, REPUL, WZPEL
**Phenolic terpenes**										
130	C_20_H_26_O_5_	Rosmanol	63.494	[M+H]^+^	346.1780	347.1853	347.1868	4.3	301, 241, 231	* URHS
**Tyrosols**										
131	C_14_H_20_O_8_	Hydroxytyrosol 4-*O*-glucoside	18.019	** [M-H]^−^	316.1158	315.1085	315.1084	−0.3	153, 123	* WZS, REPEL, URHS
132	C_17_H_24_O_11_	Oleoside 11-methylester	18.842	** [M-H]^−^	404.1319	403.1246	403.1246	0.0	223, 165	* RHPEL, REPEL, RHS, URHPUL, WZPEL
133	C_24_H_30_O_13_	Demethyloleuropein	23.000	** [M-H]^−^	526.1686	525.1613	525.1609	−0.8	495	* RHPEL, REPEL, REPUL, RES, RHS, URHPEL, URHS, WZPEL
134	C_10_H_12_O_4_	3,4-DHPEA-AC	37.593	** [M-H]^−^	196.0736	195.0663	195.0659	−2.1	135	* RES, REPEL, REPUL, RHS, URHPEL, WZPEL, WZPUL, WZS

** denotes that compounds were detected in both negative [M-H]^−^ and positive [M+H]^+^ mode of ionization while only single mode data was presented. Avocado samples mentioned in abbreviations are REPEL (Reed peel), REPUL (Reed pulp), RES (Reed seed), RHPEL (ripe Hass peel), RHPUL (ripe Hass pulp), RHS (ripe Hass seed), URHPEL (unripe Hass peel), URHPUL (unripe Hass pulp), URHS (unripe Hass seed), WZPEL (Wurtz peel), WZPUL (Wurtz pulp) and WZS (Wurtz seed). The symbol * denotes that the corresponding row’s data was obtained from that particular indicated sample.

## Data Availability

Data is contained within the article.

## Supplementary Materials

The following supporting information can be downloaded at: https://www.mdpi.com/article/10.3390/antiox12010185/s1, Figure S1: LC-ESI-QTOF-MS/MS basic peak chromatograph (BPC) for characterization of phenolic compounds of avocados; (1a) Unripe Hass Peel in negative ionization mode; (1b) Unripe Hass Peel in positive ionization mode; (2a) Ripe Hass Peel in negative ionization mode; (2b) Ripe Hass Peel in positive ionization mode; (3a) Reed Peel in negative ionization mode; (3b) Reed Peel in positive ionization mode; (4a) Wurtz Peel in negative ionization mode; (4b) Wurtz Peel in positive ionization mode; (5a) Unripe Hass Seed in negative ionization mode; (5b) Unripe Hass Seed in positive ionization mode; (6a) Ripe Hass Seed in negative ionization mode; (6b) Ripe Hass Seed in positive ionization mode (7a) Reed Seed in negative ionization mode; (7b) Reed Seed in positive ionization mode; (8a) Wurtz Seed in negative ionization mode; (8b) Wurtz Seed in positive ionization mode; (9a) Unripe Hass Pulp in negative ionization mode; (9b) Unripe Hass Pulp in positive ionization mode; (10a) Ripe Hass Pulp in negative ionization mode; (10b) Ripe Hass Pulp in positive ionization mode; (11a) Reed Pulp in negative ionization mode; (11b) Reed Pulp in positive ionization mode; (12a) Wurtz Pulp in negative ionization mode; (12b) Wurtz Pulp in positive ionization mode.
