# Supplementary material for: Phenolic Compounds Profiling and Their Antioxidant Capacity in the Peel, Pulp, and Seed of Australian Grown Avocado
Source: Antioxidants (Basel). 2023 Jan 12;12(1):185. doi: 10.3390/antiox12010185 (PMC9855119; doi:10.3390/antiox12010185)
Supplement: Supplementary file 1 [file antioxidants-12-00185-s001.zip › antioxidants-2119269-supplementary.pdf]

1a. Unripe Hass Peel (Negative)

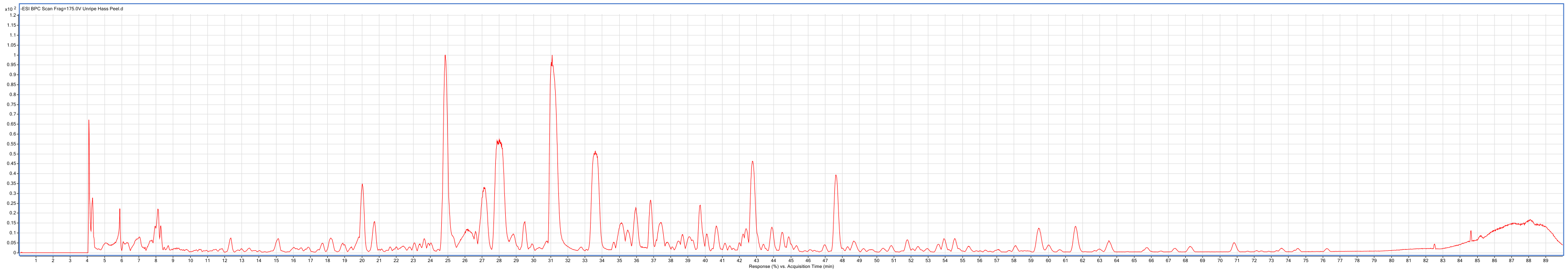

1b. Unripe Hass Peel (Positive)

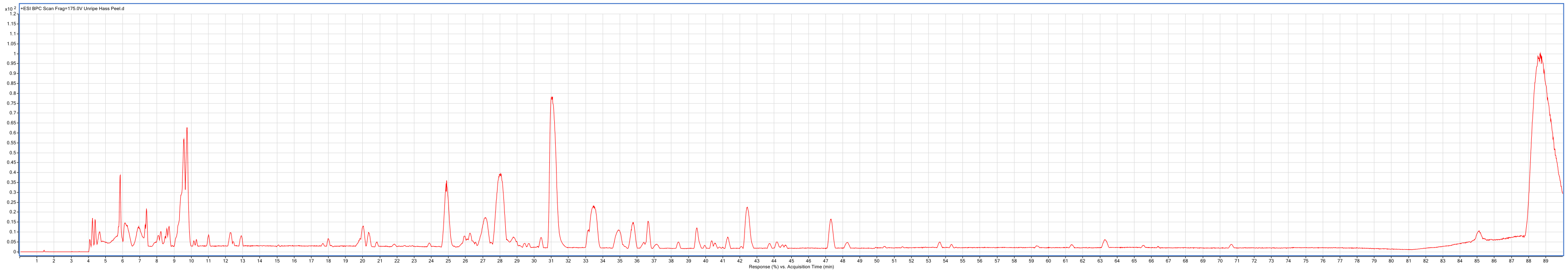

2a. Ripe Hass Peel (Negative)

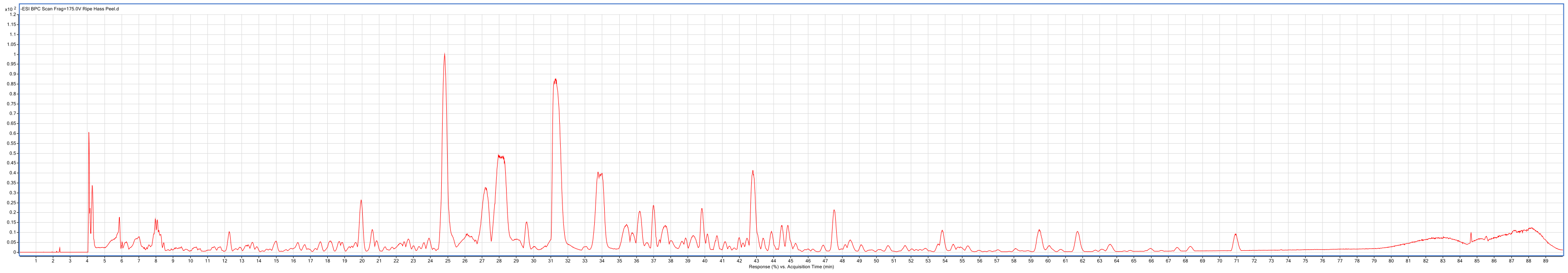

2b. Ripe Hass Peel (Positive)

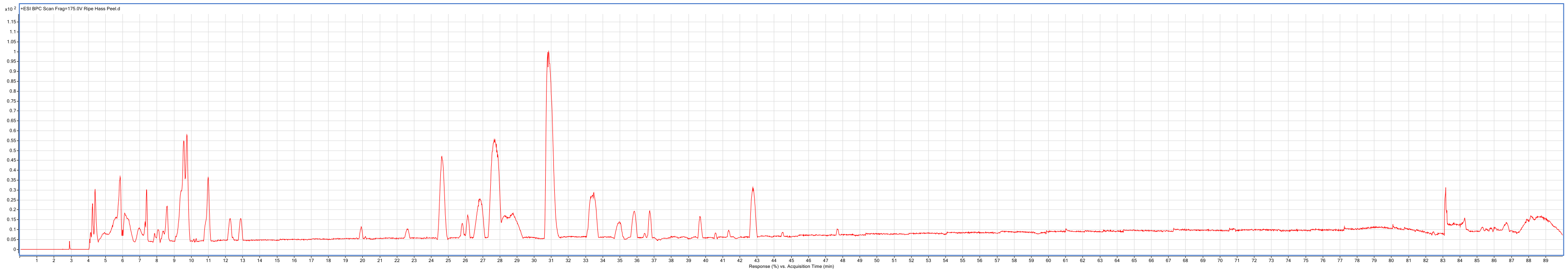

3a. Reed Peel (Negative)

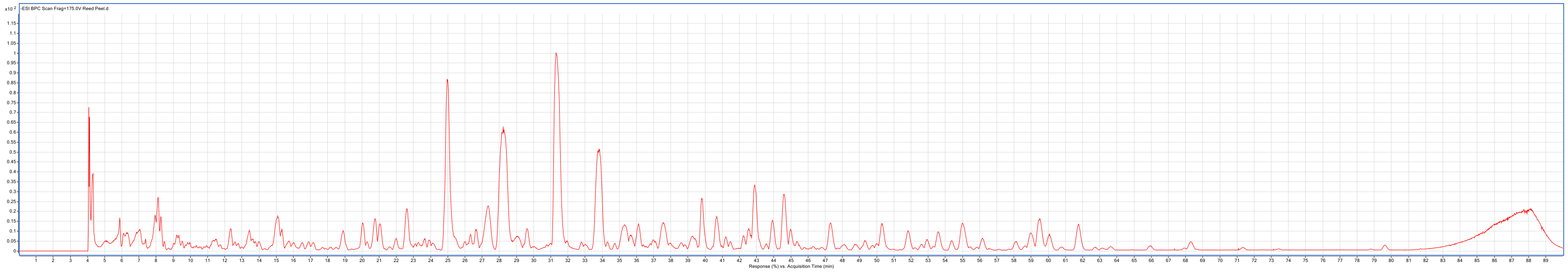

3b. Reed Peel (Positive)

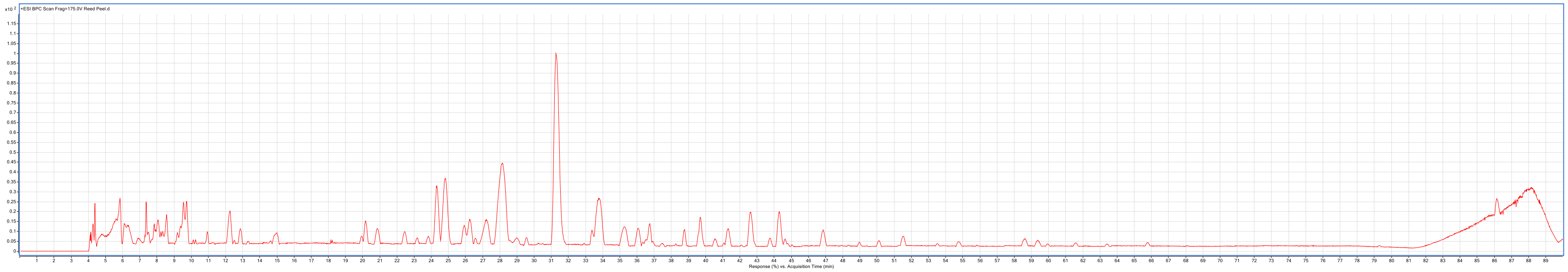

4a. Wurtz Peel (Negative)

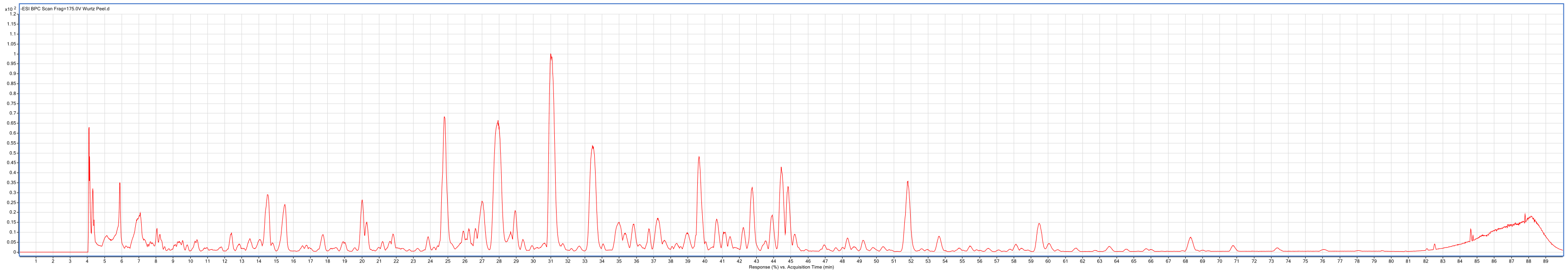

4b. Wurtz Peel (Positive)

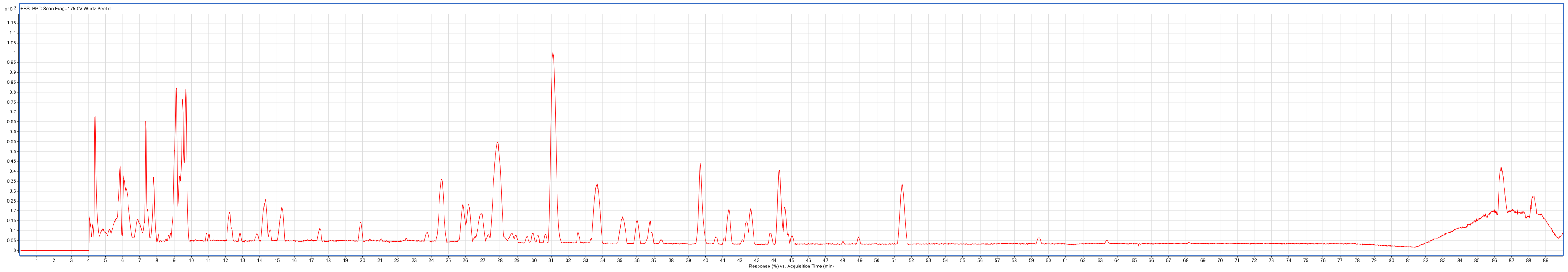

5a. Unripe Hass Seed (Negative)

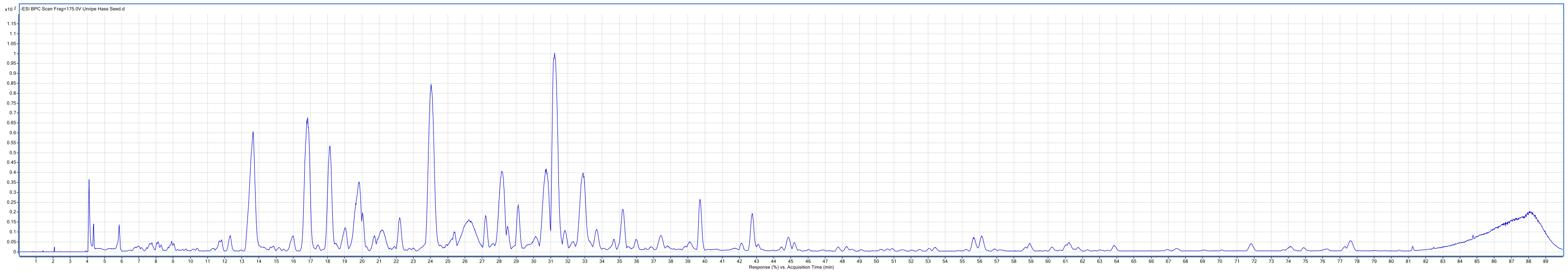

5b. Unripe Hass Seed (Positive)

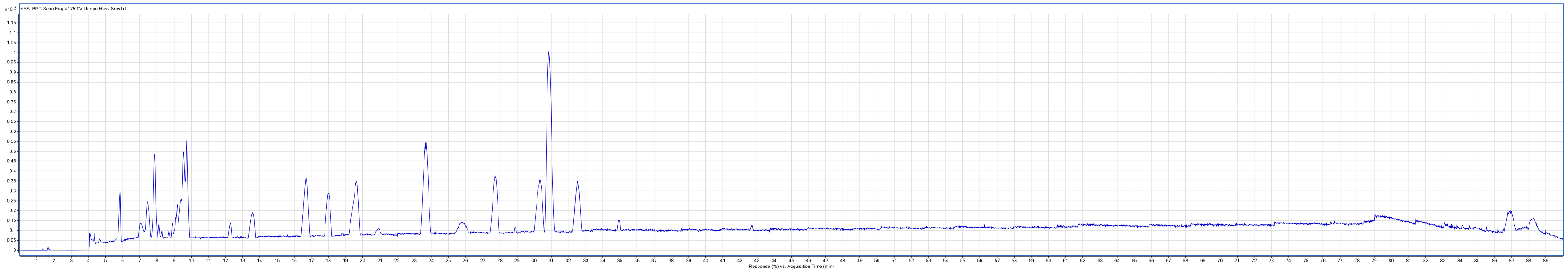

6a. Ripe Hass Seed (Negative)

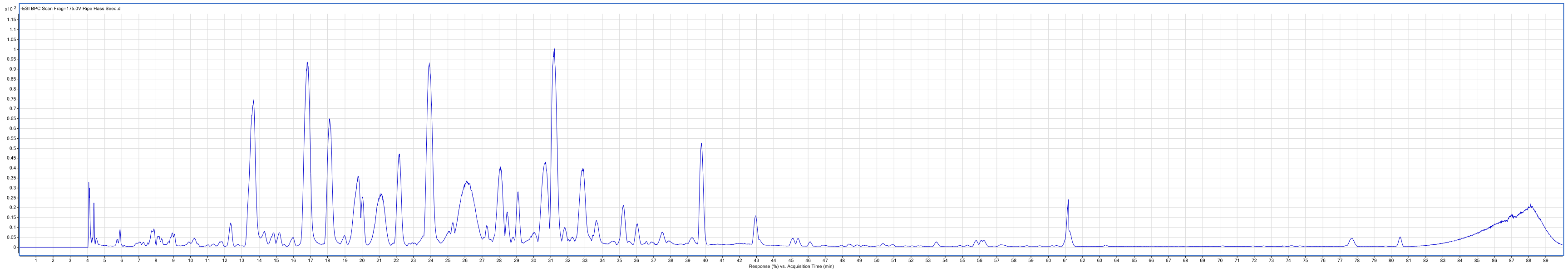

6b. Ripe Hass Seed (Positive)

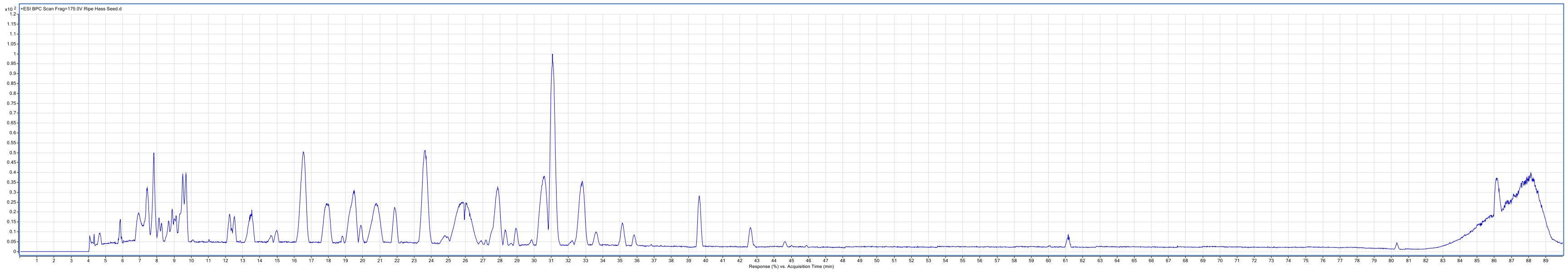

7a. Reed Seed (Negative)

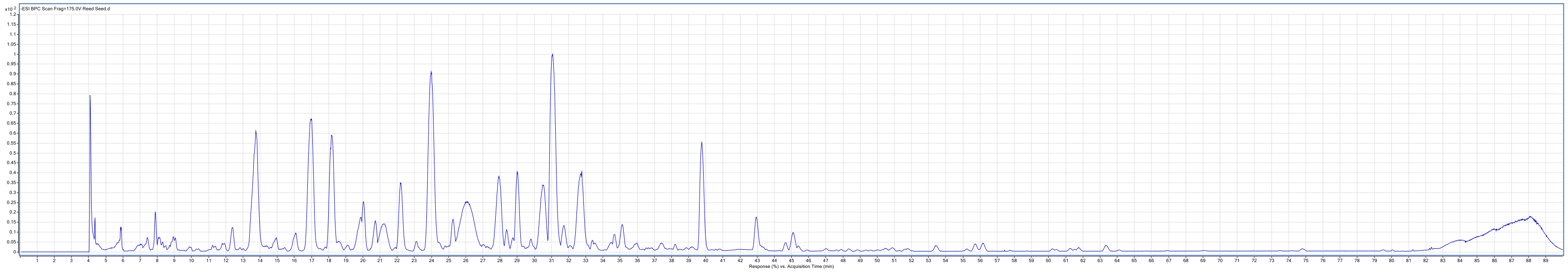

7b. Reed Seed (Positive)

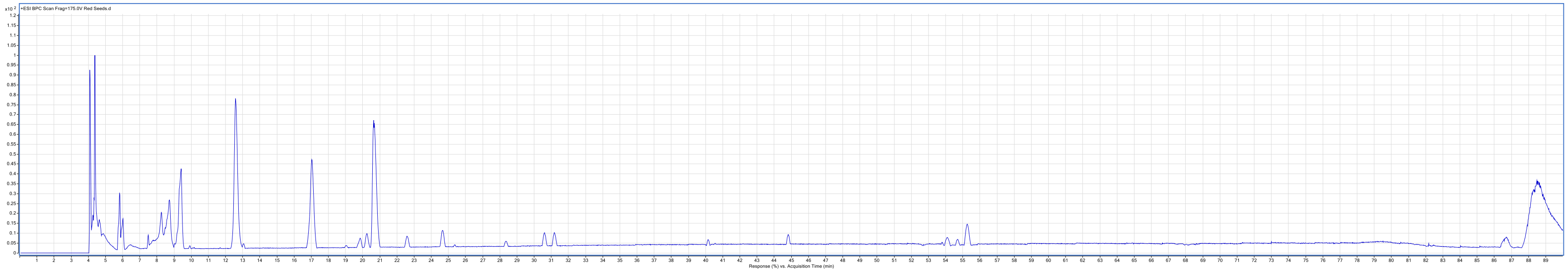

8a. Wurtz Seed (Negative)

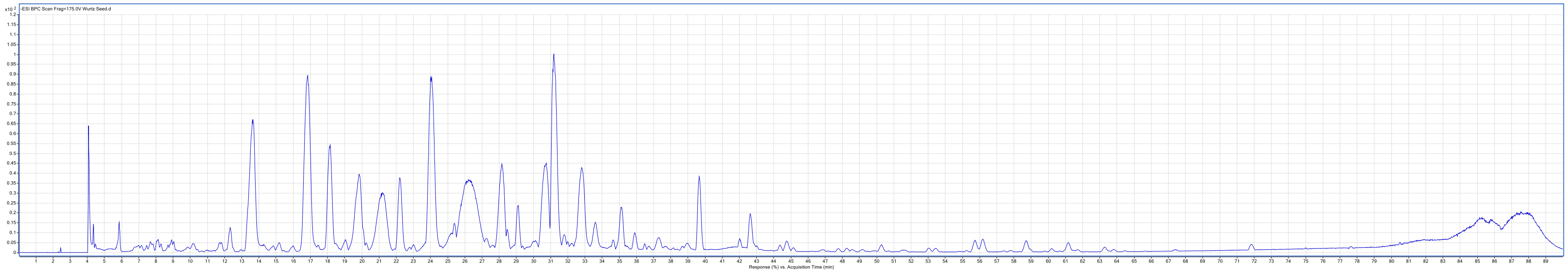

8b. Wurtz Seed (Positive)

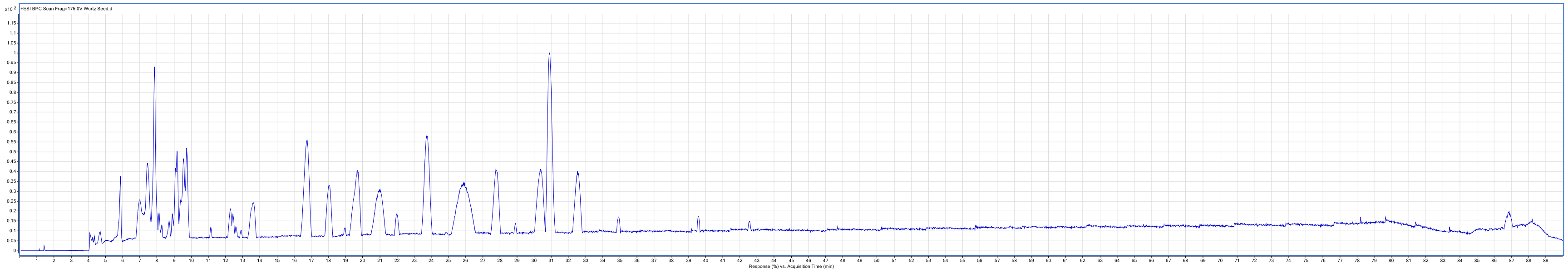

9a. Unripe Hass Pulp (Negative)

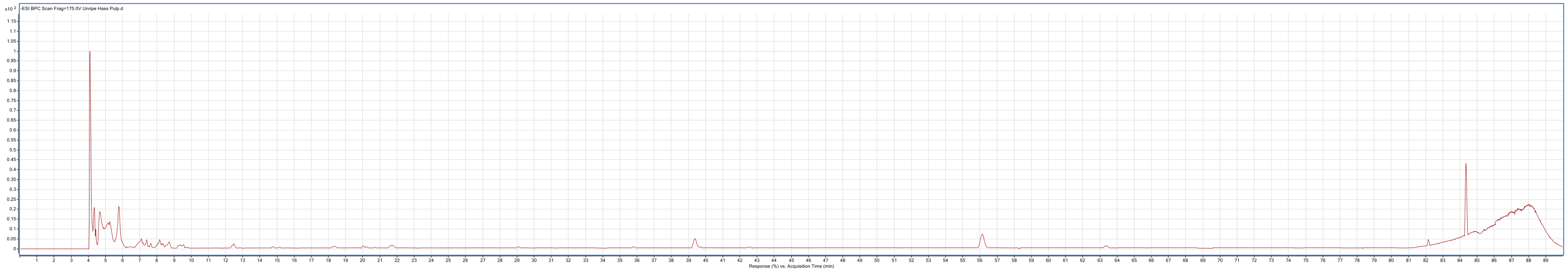

9b. Unripe Hass Pulp (Positive)

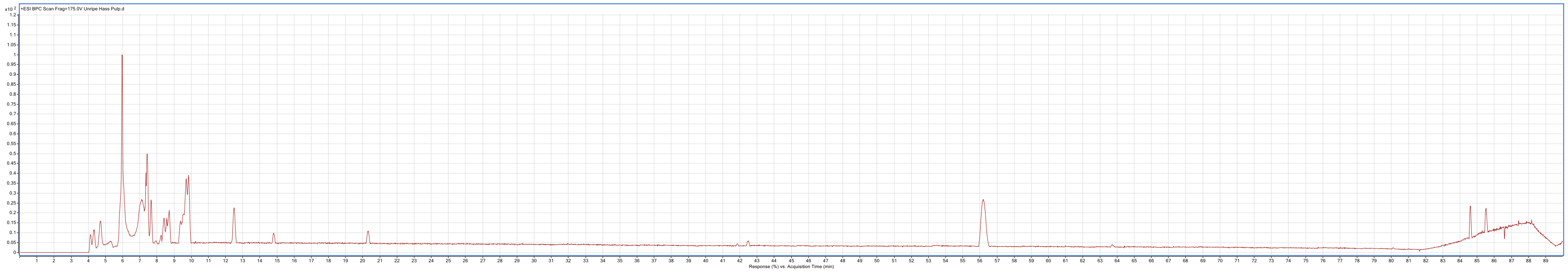

10a. Ripe Hass Pulp (Negative)

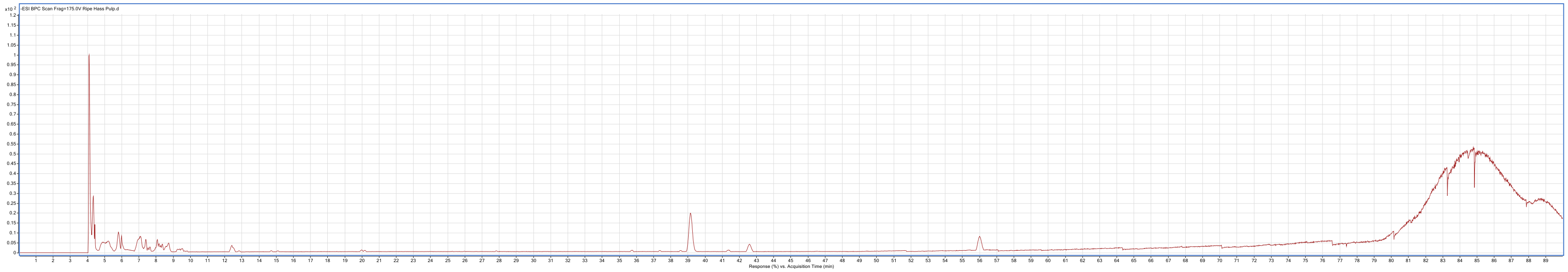

10b. Ripe Hass Pulp (Positive)

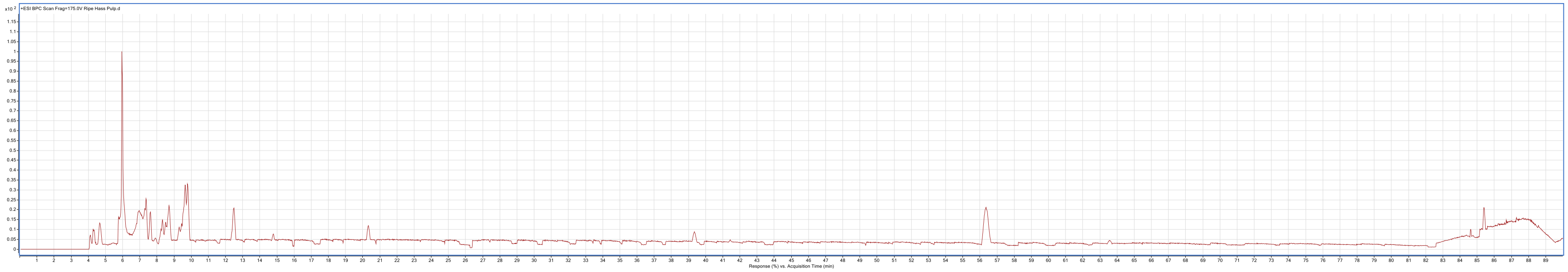

11a. Reed Pulp (Negative)

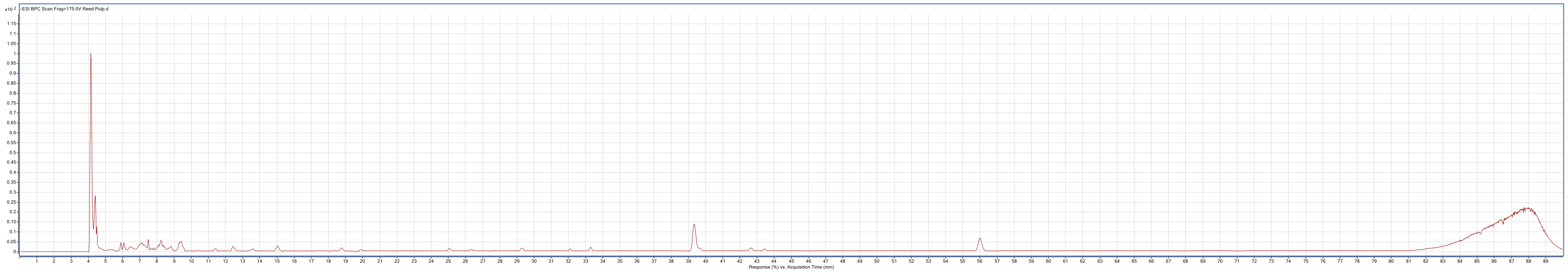

11b. Reed Pulp (Positive)

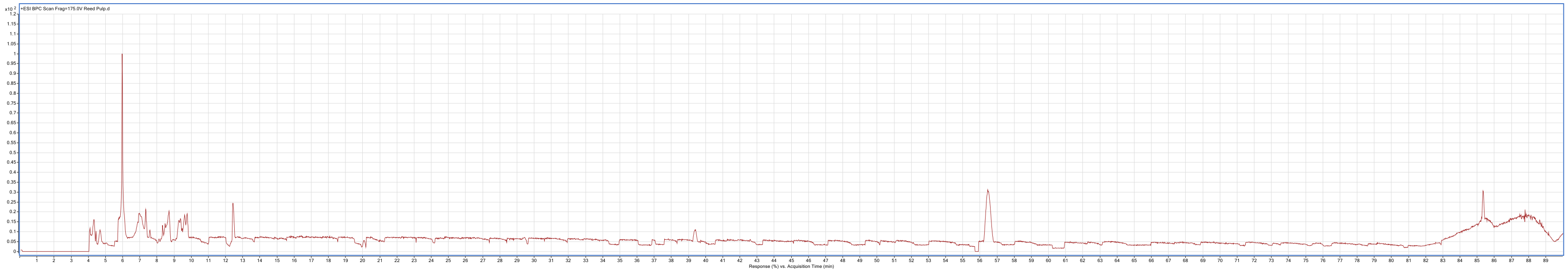

12a. Wurtz Pulp (Negative)

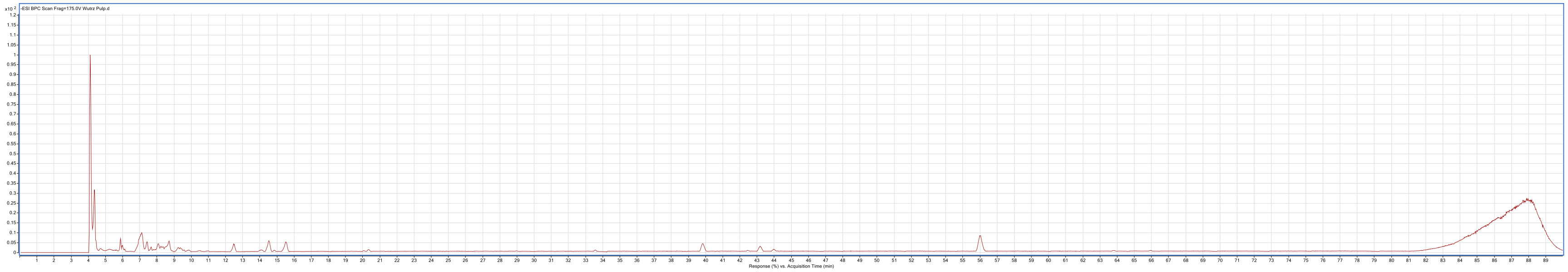

12b. Wurtz Pulp (Positive)

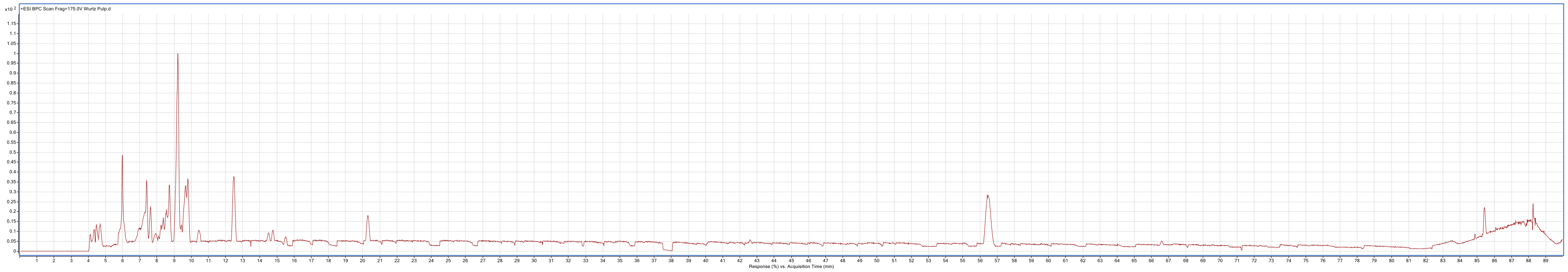

Figure S1: LC-ESI-QTOF-MS/MS basic peak chromatograph (BPC) for characterization of phenolic compounds of avocados; **(1a)** Unripe Hass Peel in negative ionization mode; **(1b)** Unripe Hass Peel in positive ionization mode; **(2a)** Ripe Hass Peel in negative ionization mode; **(2b)** Ripe Hass Peel in positive ionization mode; **(3a)** Reed Peel in negative ionization mode; **(3b)** Reed Peel in positive ionization mode; **(4a)** Wurtz Peel in negative ionization mode; **(4b)** Wurtz Peel in positive ionization mode; **(5a)** Unripe Hass Seed in negative ionization mode; **(5b)** Unripe Hass Seed in positive ionization mode; **(6a)** Ripe Hass Seed in negative ionization mode; **(6b)** Ripe Hass Seed in positive ionization mode **(7a)** Reed Seed in negative ionization mode; **(7b)** Reed Seed in positive ionization mode; **(8a)** Wurtz Seed in negative ionization mode; **(8b)** Wurtz Seed in positive ionization mode; **(9a)** Unripe Hass Pulp in negative ionization mode; **(9b)** Unripe Hass Pulp in positive ionization mode; **(10a)** Ripe Hass Pulp in negative ionization mode; **(10b)** Ripe Hass Pulp in positive ionization mode; **(11a)** Reed Pulp in negative ionization mode; **(11b)** Reed Pulp in positive ionization mode; **(12a)** Wurtz Pulp in negative ionization mode; **(12b)** Wurtz Pulp in positive ionization mode.
